# Nanoliposome-mediated delivery of sulforaphane suppresses Ehrlich ascites carcinoma growth and improves liver integrity and therapeutic outcomes in a murine model

**DOI:** 10.1186/s12885-025-15279-2

**Published:** 2025-11-25

**Authors:** Ahmad Najem Alshammari, Mohammed Saleh Alfawaz, Yusef Muhana Alenezi, Ayat B. Al-Ghafari, Huda A. Al Doghaither, Ekramy M. Elmorsy, Marwa Mahmoud Fawzy Atta, Ali Ali El-Raghi

**Affiliations:** 1 Department of Medical Laboratory Technology, College of Applied Medical Sciences, Northern Border University, Arar, 73213 Saudi Arabia; 2 Family and community department, Faculty of Medicine, Northern border University, Arar, 73213 Saudi Arabia; 3 Department of Biochemistry, Faculty of Science, King Abdulaziz University, Jeddah, 21589 Saudi Arabia; 4 Experimental Biochemistry Unit, King Fahd Medical Research Center, King Abdulaziz University, Jeddah , 21589 Saudi Arabia; 5 Center for Health Research, Northern Border University, Arar, 73213 Saudi Arabia; 6 Department of Biochemistry, Faculty of veterinary medicine, Egyptian Chinese University, Cairo, Egypt; 7 Department of Animal, Poultry, and Fish Production, Faculty of Agriculture, Damietta University, New Damietta, 34517 Egypt

**Keywords:** Sulforaphane, Nanoliposomes, Ehrlich ascites carcinoma, Oxidative stress, Apoptosis modulation

## Abstract

The anticancer activity of sulforaphane (SFN) involves multiple signaling pathways, but its mechanism against Ehrlich Ascites Carcinoma (EAC) in mice remains unclear. This study evaluated the antitumor efficacy of SFN-loaded nanoliposomes (SFN-NLPs). Ninety female Swiss albino mice were allocated into six groups: (1) untreated control, (2) SFN alone (50 mg/kg/day, orally), (3) SFN-NLPs alone (50 mg/kg/day, orally), (4) EAC-bearing mice (2.5 × 10⁶ cells, intraperitoneally), (5) EAC + SFN, and (6) EAC + SFN-NLPs. with treatment lasting 20 days in tumor-bearing groups. SFN-NLPs significantly reduced EAC tumor volume, viable cell count, and tumor marker levels, while prolonging survival more effectively than free SFN. SFN-NLPs modulated key molecular pathways by suppressing inflammatory gene expression (*NF-κB*, *COX-2*) and shifting the balance toward apoptosis via upregulation of *TP53* and *Bax* and concomitant downregulation of *Bcl-2*. Regarding liver integrity, SFN-NLPs significantly reduced DNA fragmentation and hepatic oxidative stress, while modulating systemic inflammation through reductions in total leukocyte count, TNF-α, and C-reactive protein. In addition, SFN-NLPs conferred superior protection against both histopathological and ultrastructural liver damage. Furthermore, molecular docking analysis suggested plausible interactions of SFN with proteins associated with antioxidant defense, inflammation regulation, and apoptosis, indicating a potential modulatory role. In conclusion, nanoformulated SFN enhances stability, augments efficacy, and facilitates sustained release and bioavailability, thereby strengthening its antitumor, anti-inflammatory, antioxidant, and pro-apoptotic effects in EAC-bearing mice. These findings highlight the potential of SFN-NLPs to suppress tumor growth by modulating inflammatory and apoptotic gene expression while preserving liver integrity.

## Introduction

Cancer represents a critical global health issue, imposing extensive societal burdens. As the second most common cause of death, it compromises the function of virtually every tissue and organ in the body [1]. The ability of malignant cells to proliferate uncontrollably and metastasize enables tumors to affect distant organs, resulting in considerable morbidity and mortality [2, 3]. Humans are continuously exposed to diverse carcinogenic agents throughout life. These agents may be endogenous, including genetic mutations, immunologic disturbances, or hormonal imbalances, or exogenous, such as physical, chemical, environmental, and biological factors, in addition to lifestyle and dietary influences [4]. The Ehrlich ascites carcinoma (EAC) model is widely applied in vivo for research on tumor biology and for evaluating immunomodulatory and anticancer therapies [5, 6]. While primarily confined to the abdominal cavity, EAC cells are capable of spreading to distant organs, resulting in tissue damage and compromised organ function [7, 8]. The rapid growth of EAC profoundly impacts the host immune system, disrupting immune cell functions and enabling unchecked tumor growth and metastasis [9]. These effects highlight the potential value of therapeutic approaches that restore immune competence in EAC-bearing individuals [10]. Current approaches for treating malignant tumors include radiotherapy, surgery, and chemotherapy, with chemotherapeutic agents offering substantial therapeutic benefits. Nonetheless, their effectiveness is often hindered by the emergence of resistance and serious side effects, which may compromise overall treatment outcomes [11, 12]. Thus, the search for innovative anticancer therapies remains an important research goal. Natural or synthetic chemopreventive compounds represent a promising strategy to prevent and manage cancer [13, 14].

Cruciferous vegetables are abundant in chemoprotective compounds, and their dietary intake has been associated with reduced cancer risk, especially during early carcinogenesis, and may provide ongoing protection as tumors progress [14, 15]. Cabbage (Brassica oleracea), a prominent member of the cruciferous vegetable family, is utilized both as a dietary staple and in traditional medicine for conditions like headaches, diarrhea, gout, and peptic ulcers [16, 17]. Its chemopreventive properties are mainly due to phytochemicals including indoles, indole-3-carbinol, and SFN [18]. SFN, present at approximately 7.58 µg/g DW in cabbage, demonstrates anticancer activities through multiple mechanisms: inhibiting histone deacetylases [19], alleviating oxidative stress [16, 17], modulating transcription factors and signaling cascades [20], inducing apoptosis and cell cycle arrest [21], and suppressing inflammation via Cox-2 and NF-κB pathways [22]. Overall, these findings highlight SFN’s capacity as a natural anticancer agent, capable of reducing tumor growth, immune dysfunction, oxidative damage, and inflammation in both EAC and other cancer types.

Despite the promising anticancer properaties of SFN, its clinical utility is restricted due to rapid degradation and low stability, as is typical for many natural compounds [23]. The use of nanocarriers, particularly liposomal systems, can address these issues by enhancing drug stability, retention, and targeted delivery to pathological tissues [24]. SFN encapsulation has been shown to improve its poor stability [25], augment efficacy, facilitate sustained release [26] and enhance anticancer potency in melanoma cells [27], alongside anti-inflammatory efficacy in models of acute and chronic rheumatoid arthritis [28]. Liposomes are organic nanocarriers characterized by strong hydrophilic, high biocompatibility and the ability to encapsulate both hydrophobic and hydrophilic drugs [29]. In addition, it was demonstrated that functionalized liposomes promote medications accumulation in tumors by improving permeability and retention through enhanced permeability and retention (EPR) effects [30]. Also, biodegradable nanoparticles were shown to effectively regulate drug release and enhance tumor cell uptake [31]. Despite heightened interest in SFN’s pharmacological properties, in vivo studies in the EAC model remain relatively few. To our knowledge, this is the first study to evaluate SFN-loaded nanoliposomes (SFN-NLPs) in this model, focusing on their potential apoptotic effects on tumor cells and protective effects on hepatic tissue.

## Materials and methods

### Molecular docking

Molecular docking studies were performed to evaluate the interactions between SFN and various target proteins. AutoDock Vina, a widely utilized docking tool employing a scoring mechanism, was used to conduct docking simulations aimed at predicting binding conformations and affinities. Protein structures were obtained from the Protein Data Bank (PDB) and subsequently processed by removing water molecules and adding hydrogens to ensure accurate modeling of interactions. AutoDock Vina was utilized to create the ligand SFN, which was subsequently positioned in the active sites of the target proteins. The resulting docking poses were analyzed to identify optimal binding conformations, hydrogen bonds, and hydrophobic interactions. This provided insight into the potential mechanisms by which SFN could inhibit or modify these targets.

### Preparation of SFN- loaded nanoliposomes (SFN-NLPs)

SFN with ≥ 98% purity was sourced from Sigma-Aldrich (USA). Cholesterol and L-α-phosphatidylcholine (L-PC) were obtained from Avanti Polar Lipids (USA). The full composition of liposomes was: 70 mg L-PC (0.09 mmol) and 30 mg cholesterol (0.078 mmol), giving a total lipid concentration of 10 mg/mL after hydration.

The SFN-loaded liposomes were prepared using the thin-film hydration technique. L-PC and cholesterol were dissolved in 10 mL chloroform/methanol (2:1 v/v) in a round-bottom flask. The organic solvent was evaporated under vacuum using a rotary evaporator to form a thin lipid film. Hydration of the lipid film was performed using 5 mL PBS (pH 7.4) containing 1 mg SFN (≈ 5 µmol), corresponding to a final concentration of 200 µg/mL SFN in the hydration solution. This method ensures incorporation of the hydrophobic SFN within the lipid bilayer rather than in the aqueous core. The mixture was incubated at 37 °C with gentle agitation for 30 min. Multilamellar vesicles were converted into small unilamellar vesicles via probe sonication (SONICS Vibra Cell, USA) at 30% amplitude in 10-second on/off cycles for 10 min in an ice bath to prevent thermal degradation. Unencapsulated SFN was removed by centrifugation at 15,000 × g for 30 min, and the supernatant containing the encapsulated liposomes was harvested. Zeta potential, polydispersity index (PDI), and particle size of SFN-loaded nanoliposomes were measured using a Zetasizer Nano ZS. Distilled water was used as a diluent for Z-average determination, and zeta potential values were recorded in triplicate. Morphology of nanoparticles was examined using transmission electron microscopy (TEM; JEOL 2100, Japan) at 160 kV.Drug loading and encapsulation efficiency were determined by quantifying free SFN in the supernatant using UV–Vis at 260 nm:

- Drug loading (%) = [Encapsulated SFN/Total liposome mass] × 100.
- Encapsulation efficiency (%) = [Encapsulated SFN/Total SFN added] × 100.

This preparation method ensures SFN, a hydrophobic compound, is primarily partitioned into the lipid bilayer, taking advantage of its hydrophobicity for stable incorporation into the nanoliposome structure.

In addition, in vitro drug release of SFN-NLPs was evaluated under simulated physiological conditions (PBS, pH 7.4, 37 °C) to investigate sustained-release behavior. Storage stability was assessed over 1–4 weeks at 4 °C and 25 °C by monitoring changes in particle size, PDI, zeta potential, and drug leakage to ensure compliance with clinical storage requirements. Furthermore, the release and stability profiles were experimentally confirmed. The in vitro release study demonstrated a biphasic pattern characterized by an initial burst phase during the first 6 h, followed by a sustained and gradual release up to 72 h, indicating the prolonged retention of SFN within the lipid bilayer. The storage stability test revealed negligible changes in particle size, PDI, and zeta potential values over four weeks at 4 °C, with minimal drug leakage (< 5%), confirming the physicochemical stability of the nanoliposomes under refrigerated conditions.

### Collection and viability assessment of EAC cells

The Ehrlich Ascites Carcinoma (EAC) cell line was procured from the National Cancer Institute (NCI), Cairo, Egypt, and maintained in vivo by serial intraperitoneal passage of 2.5 × 10⁶ cells into female Swiss albino mice at 10-day intervals, according to the method described by Yılmaz et al. [32]. To induce anesthesia, a ketamine–xylazine mixture (80 mg/kg and 10 mg/kg, respectively) was administered intraperitoneally. On days 7 or 8 after EAC inoculation, ascitic fluid was collected from mice while maintaining the same anesthetic procedure. Collected ascitic fluid was subjected to viability testing by hemocytometer counting with 0.4% trypan blue dye exclusion. The suspension was then standardized to 2.5 × 10⁶ viable cells in 0.2 mL PBS for inoculation.

### Animal handling and experimental protocol

Healthy female Swiss albino mice (*n* = 90; average body weight, 19.2 g) were obtained from the Faculty of Medicine, Mansoura University, Egypt. They were housed in groups in standard plastic cages and maintained under controlled environmental conditions: relative humidity 45%, temperature 25 °C, and a 12/12 h light-dark cycle. Throughout the study, mice had unrestricted access to food and water. Before initiating the experiments, the animals were acclimated to the laboratory environment for one week. All procedures were performed in accordance with the *International Guiding Principles for Biomedical Research Involving Animals* (1985) and the *8th edition of the Guide for the Care and Use of Laboratory Animals* (NRC, 2011). A total of 90 female Swiss albino mice were randomly assigned into six groups of 15 animals each: (1) untreated control, (2) SFN alone (50 mg/kg/day, orally), based on the safety and effective dose reported by Abouzed et al. (2021), (3) SFN-loaded nanoliposomes (SFN-NLPs) alone (50 mg/kg/day, orally), (4) EAC-bearing mice inoculated intraperitoneally with 2.5 × 10⁶ cells on day 1 and left untreated for 20 days, (5) EAC + SFN, and (6) EAC + SFN-NLPs. Treatments in tumor-bearing groups were administered daily for 20 days. Animal body weights were measured at the start of the study and at its conclusion. Daily observations were conducted to assess survival in EAC-bearing mice. Of the 15 mice per group, 7 mice were allocated for biochemical analyses, tissue collection, histopathological examinations, and molecular assessments. Some mortality occurred in EAC-bearing groups during the study, and these instances were recorded and accounted for in survival analyses.

### Sample collection

Seven mice per experimental group were sacrificed via cervical dislocation at the conclusion of the study. To ensure uniformity in blood sampling, all animals underwent a 10-hour fast and were anesthetized with inhaled isoflurane to reduce distress. Blood was drawn through retro-orbital venous sinus puncture and separated into two fractions: one in an EDTA tube for white blood count analysis and the other in a plain tube for serum isolation. The number of white blood cells (WBCs) was measured by the improved Neubauer method employing a hemocytometer. Hayem’s solution was used for counting erythrocytes, whereas Turk’s solution was applied to stain and enumerate leukocytes. The serum was preserved under proper conditions for subsequent biochemical and inflammatory cytokine assays.

### Serum biochemical analysis

Biochemical analyses of serum samples were performed using standardized commercial kits according to the manufacturers’ protocols. The assessed parameters included total protein (TP; Cat. No. CBP007-K), albumin (Cat. No. MET-5017), alanine aminotransferase (ALT; Cat. No. MET-5123), aspartate aminotransferase (AST; Cat. No. MET-5127), alkaline phosphatase (ALP; Cat. No. CBA-301), and lactate dehydrogenase (LDH; Cat. No. MAK066). Globulin levels were derived by subtracting albumin from the total protein concentration. Commercial ELISA kits (Cat. No. MBS267737; MyBioSource, San Diego, USA) were used to determine serum TNF-α concentrations, with all procedures performed according to the manufacturer’s guidelines. High-sensitivity C-reactive protein (hs-CRP) levels were determined using a mice CRP ELISA kit (eBioscience, Inc.), also following the supplied protocol. Serum alpha-fetoprotein (AFP) levels were measured using an automated enzyme-linked fluorescent assay (ELFA) on the mini-VIDAS^®^ AFP system (bioMérieux, Marcy-l’Étoile, France), which is validated for mouse serum with a detection range of 0.5–100 ng/mL. Serum concentrations of carcinoembryonic antigen (CEA) were quantified using the Mouse CEA ELISA Kit (MyBioSource, San Diego, USA), validated for mouse serum with a detection range of 0.2–50 ng/mL. Serum levels of cancer antigen 19 − 9 (CA 19 − 9), cancer antigen 125 (CA-125), and cancer antigen 15 − 3 (CA 15 − 3) were measured using Mouse CA19-9, CA-125, and CA15-3 ELISA Kits (MyBioSource, San Diego, USA), with detection ranges of 1–200 U/mL, 0.5–100 U/mL, and 1–150 U/mL, respectively.

### Evaluation of tumor burden and viable cell numbers in Ascites

Ascitic fluid was collected from each mouse after EAC inoculation and centrifuged at 1200 rpm for five minutes. The pellet of tumor cells was measured using a graduated conical centrifuge tube, and cell viability was determined by the Trypan blue exclusion method, with viable cells remaining unstained and non-viable cells appearing blue.

### Antioxidant status

After dissection, liver tissues were gently rinsed with ice-cold 1.15% potassium chloride (KCl) solution to remove residual blood and debris. Samples intended for biochemical analysis were immediately frozen at − 20 °C, while representative sections were fixed in 10% neutral-buffered formalin for subsequent histopathology. Following homogenization of liver tissues in ice-cold phosphate buffer (pH 7.4), samples were centrifuged at 4000 rpm for 15 min at 4 °C to obtain clear supernatants. These supernatants were subsequently used to assess oxidative stress markers and key antioxidant enzyme activities. Specifically, levels of superoxide dismutase (SOD; Cat. No. SD 2521), catalase (CAT; Cat. No. CA 2517), glutathione peroxidase (GPx; Cat. No. GP 2524), and reduced glutathione (GSH; Cat. No. GR 2511) were quantified. Levels of malondialdehyde (MDA), an indicator of lipid peroxidation, were determined using the TBARS method (Cat. No. MD 2529). Commercial colorimetric kits (Biodiagnostic Co., Egypt) were used in accordance with the manufacturer’s guidelines. The generation of reactive oxygen species (ROS) was quantified using a fluorometric assay based on the oxidation of 2′,7′-dichlorodihydrofluorescein diacetate (DCFH-DA). Supernatants were incubated with 10 µM DCFH-DA for 30 min at 37 °C in the dark, and fluorescence was measured at 485/530 nm with a microplate reader, following Wang and Joseph [33].

### DNA damage biomarkers

The extent of DNA fragmentation in Ehrlich Ascites Carcinoma (EAC) cells was quantified using a commercially available colorimetric ELISA kit, adhering to the manufacturer’s guidelines. Ascetic fluid from EAC-treated mice was collected, and cells were pelleted by centrifugation at 1200 rpm for 5 min, followed by washing with Phosphate-Buffered Saline. Following treatment with the detection reagent, the color change was developed and absorbance recorded at 405 nm on a microplate reader, and the DNA fragmentation was expressed as a percentage relative to control cells.

### Molecular analysis via real-time PCR

RNA was isolated from tumor cell of mice bearing ESC using as the initial step of RT-PCR. Tissue samples were lysed in 1 mL QIAzol Lysis Reagent and homogenized using a Tissuelyser II. Chloroform was then added, and samples were centrifuged at 12,000 × g for 15 min. The resulting aqueous phase was carefully collected, and RNA was precipitated by the addition of isopropanol. Following isopropanol precipitation, RNA samples were centrifuged at 12,000 × g for 10 min to collect the pellet. The isopropanol was removed, and the pellet was rinsed with ethanol. A second centrifugation at 7,500 × g for 5 min ensured the removal of ethanol traces. The RNA was then dissolved in DNase/RNase-free water, and its quantity was assessed. Complementary DNA (cDNA) was generated from total RNA in the second stage of RT-PCR using the iScript™ cDNA Synthesis Kit per the manufacturer’s instructions. In the subsequent stage, cDNA samples were combined with iTaq Universal SYBR Green Supermix (Bio-Rad, Cat. No. 172–5121) for amplification. Specific primers targeting apoptosis-related genes, including tumor Protein p53 (p53), Bcl-2 Associated X Protein (Bax), B-cell lymphoma 2 (Bcl-2), nuclear factor kappa B (NF-κB), cyclooxygenase-2 (Cox-2), and tumor necrosis factor alpha (*TNF-α*) were adopted from studies of Mohamed et al. [34] and Abouzed et al. [14] and synthesized by Macrogen (Seoul, South Korea). The primers were reconstituted in nuclease-free water to prevent RNA or DNA degradation (Table 1). The primer sequences. Amplification was performed on a Rotor-Gene Q system (Qiagen), and relative gene expression levels were calculated using the 2^–ΔΔCt method. Melt curve analysis confirmed the specificity of amplification for all primer sets, showing single distinct peaks. In addition, the expression of the housekeeping gene β-Actin remained stable across all treatment groups, validating its use as an internal control.

**Table 1:** Sequences of primers used in qRT-PCR analysis

Gene	Sequences (5′−3′)	Amplicon size (bp)
*Bax*	F: GTCTCCGGCGAATTGGAGAT R: ACCCGGAAGAAGACCTCTCG	100
*BCL-2*	F: CATCGCCCTGTGGATGACT R: GGCCATATAGTTCCACAAAGGC	95
*TP53*	F: CCCCTGTCATCTTTTGTCCCT R: AGCTGGCAGAATAGCTTATTG	137
*NF-κB*	F: GAAATTCCTGATCCAGACAAAA R: ATCACTTCAATGGCCTCTGTGT	106
*Cox-2*	F: CAAGGGAGTCTGGAACATTG R: ACCCAGGTCCTCGCTTATGA	180
*TNF-α*	F: GACAAGGCTGCCCCGACTACG R: CTTGGGGCAGGGGCTCTTGAC	120
*β-Actin*	F: ACTATTGGCAACGAGCGGTT R: CAGGATTCCATACCCAAGAAGGA	360

*TP53 *tumor protein, *Bax Bcl-2*-associated X protein, *Bcl-2 *B-cell lymphoma 2, *NF‐κB *Nuclear factor kappa B, *Cox-2 *Cyclooxygenase-2, *TNF-α *tumor necrosis factor alpha

### Flowcytometry studies

Single-cell suspensions were generated from ascitic fluid by collecting EAC cells from the peritoneal cavity, followed by centrifugation at 300×g for 5 min at 4 °C. Mechanical disruption of solid tumor tissues was not performed. We prepared and permeabilized 1 × 10⁶ cells using the BD Cytofix/Cytoperm™ Kit (BD Biosciences, Cat# 554714) in accordance with the manufacturer’s guidelines. Cells underwent staining with Alexa Fluor^®^ 488-conjugated anti-p53 (clone DO-7, Cell Signaling Technology, Cat# 48818) and PE-conjugated anti-Ki-67 (clone SolA15, Thermo Fisher/eBioscience, Cat# 12–5698−82) for 30 min at 4 °C, protected from light. After two washes in Perm/Wash buffer, the cells were resuspended in staining buffer and analyzed with a BD FACSCalibur™ cytometer, which recorded 10,000 events per sample. Data processing was performed utilizing FlowJo™ v10 (Tree Star Inc.). The results were expressed as the percentage of positive cells and the mean fluorescence intensity (MFI). Isotype and fluorescence minus one (FMO) controls were utilized to verify the precision of the gating process.

### Flow cytometric analysis of p53 and Ki-67 expression

Single-cell suspensions were prepared from ascitic cells collected from the peritoneal cavity. Cells were fixed and permeabilized using the BD Cytofix/Cytoperm™ Kit (BD Biosciences, Cat# 554714) following the manufacturer’s instructions. Cells were stained with Alexa Fluor^®^ 488-conjugated anti-p53 (clone DO-7, Cell Signaling Technology, Cat# 48818) and PE-conjugated anti-Ki-67 (clone SolA15, Thermo Fisher/eBioscience, Cat# 12–5698−82) for 30 min at 4 °C in the dark. Following two washes in Perm/Wash buffer, the cells were resuspended in staining buffer and analyzed using a BD FACSCalibur™ cytometer, which recorded 10,000 events per sample. Data processing was conducted using FlowJo™ v10 (Tree Star Inc.). The findings were presented as the percentage of positive cells and the mean fluorescence intensity (MFI). Isotype and fluorescence minus one (FMO) controls were employed to ensure the accuracy of the gating.

### Liver histopathology

For histological analysis, tissues were harvested and immediately immersed in 10% formalin at a ratio of 20:1 (formalin to tissue volume). Following three days of fixation, the samples were dehydrated in graded alcohol solutions and cleared using xylene. The cleared tissues were infiltrated with molten paraffin for one hour, followed by preparation of paraffin blocks. Sections were cut using a rotary microtome, dehydrated in graded alcohols, stained with hematoxylin and eosin, and observed under a microscope. Hepatic steatosis was assessed semi-quantitatively following AASLD criteria as outlined in Table 2. Histopathological analysis of the liver was conducted in three mice, each slide comprising three tissue sections. Four fields per section were assessed under 400× magnification, providing 12 fields per organ. Lesions were graded semi-quantitatively on a scale from 0 (absent) to 3 (severe), and mean lesion scores were obtained by averaging all field scores for each mouse.

**Table 2:** Criteria for semi-quantitative liver histopathological analysis

Score	Composite Liver Evaluation Score
0 (none)	No pathological changes
1 (mild)	Histological examination revealed rare to mild degeneration and necrosis of hepatocytes, accompanied by minimal inflammation and sporadic vascular congestion.
2 (moderate)	Histological examination revealed moderate vacuolar degeneration, areas of hepatocellular necrosis and inflammation ranging from scattered to multifocal, and mild vascular congestion.
3 (severe)	Histological examination revealed pronounced hepatic degeneration, extensive inflammatory infiltration, necrosis, and moderate to severe congestion of blood vessels.

### Transmission Electron Microscope (TEM)

Liver tissues intended for ultra-structural analysis were fixed in 2.5% glutaraldehyde in 0.1 M phosphate buffer at 4 °C for 24 h. Samples were then thoroughly washed and post-fixed with 1% osmium tetroxide for 2 h. For ultrastructural analysis, samples were dehydrated progressively in ethanol (50–100%), cleared in acetone, and embedded in epoxy resin. Sections approximately 60–70 nm thick were cut using an ultramicrotome, stained with uranyl acetate and lead citrate, and examined under a JEOL 2100 TEM (Japan) at 160 kV.

### Statistical analysis

Normality and homogeneity of variance were verified by the Shapiro-Wilk and Levene’s tests. One-way ANOVA (SAS, 2012; Proc ANOVA) was applied for group comparisons, with Tukey’s test used for post hoc multiple comparisons. Exact *p*-values were reported for all comparisons and the unit of analysis for each figure and table was clearly specified. Survival data were analyzed using the Kaplan–Meier method, and differences among survival curves were evaluated with the log-rank (Mantel–Cox) test. Median survival times, hazard ratios, and corresponding 95% confidence intervals (CIs) were determined, with *p* < 0.05 considered statistically significant. Data are expressed as mean ± standard error, and *p* < 0.05 was considered significant. Figures were plotted with GraphPad Prism 9.0, while multivariate analyses, such as heatmap clustering and PCA, were performed using SRplot (https://www.bioinformatics.com.cn/en.

## Results

### Molecular docking

**Fig. 1:**
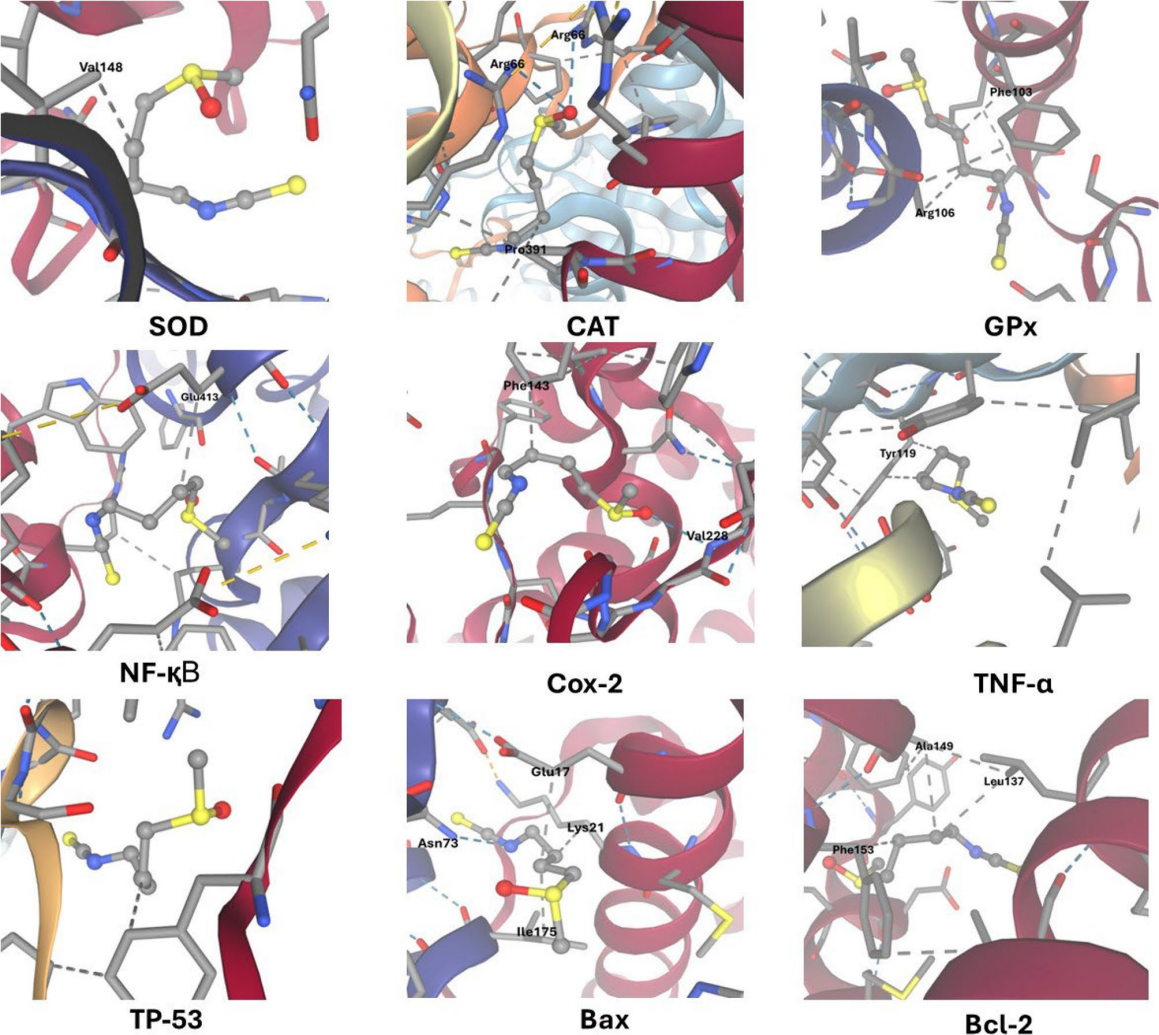
Representative figures for docking results of SFN and various target proteins showing hydrogen bonding interactions, and hydrophobic contacts represented with dotted blue and gray lines, respectively, and the interacting residues

Molecular docking studies were conducted to evaluate the potential binding interactions of SFN with various target proteins. SFN showed moderate to favorable binding affinities, with the highest predicted affinity for NF-κB (−4.5 kcal/mol), followed by COX-2 and GPx (−4.1 kcal/mol). SFN was predicted to form hydrogen bonds with residues Arg66 in catalase, Val228 in CO-2, and Asn73 in Bax, forming 2, 1, and 1 hydrogen bonds, respectively. Hydrophobic interactions were also observed, with Pro391 in catalase, Phe143 in CO-2, and Glu17, Lys21, and Ile175 in Bax contributing to the predicted binding stability. These docking results suggest that SFN may interact with functionally important regions of these proteins, indicating a potential modulatory role in relevant biological pathways (Table 3; Fig. 1).

**Table 3:** Summary of docking results showing binding affinities, hydrogen bonding interactions, and hydrophobic contacts between SFN and various target proteins. The data include the number of hydrogen bonds, key residues involved in hydrogen bonding and hydrophobic interactions, and the corresponding binding free energies (Kcal/mol)

Target	Affinity (Kcal/M)	Hydrogen bonds	Residues	Hydrophobic contacts	Residues
CAT	−3.6	2	Arg66	1	Pro391
SOD	−3.4	0	-	1	Val148
GPx	−4.1	0	-	2	Phe103, Arg106
NF-қB	−4.5	0	-	1	Glu413
TNF-α	−3.9	0	-	2	Tyr119
COX-2	−4.1	1	Val228	1	Phe143
TP-53	−3	0	-	1	Phe212
Bax	−4	1	Asn73	3	Glu17, Lys21, Ile175
Bcl-2	−3.8	0	-	3	Leu137, Ala149, Phe153

*CAT* catalase, *SOD *superoxide dismutase, *GPx *glutathione peroxidase Nuclear factor kappa B, *Cox-2* Cyclooxygenase-2, *TNF-α *tumor necrosis factor- alpha, *TP53 *Tumor Protein 53, *Bax* *Bcl-2*-associated X protein, *Bcl-2 *B-cell lymphoma 2

### Characterization of SFN-loaded nanoliposomes (SFN-NLPs)

**Fig. 2:**
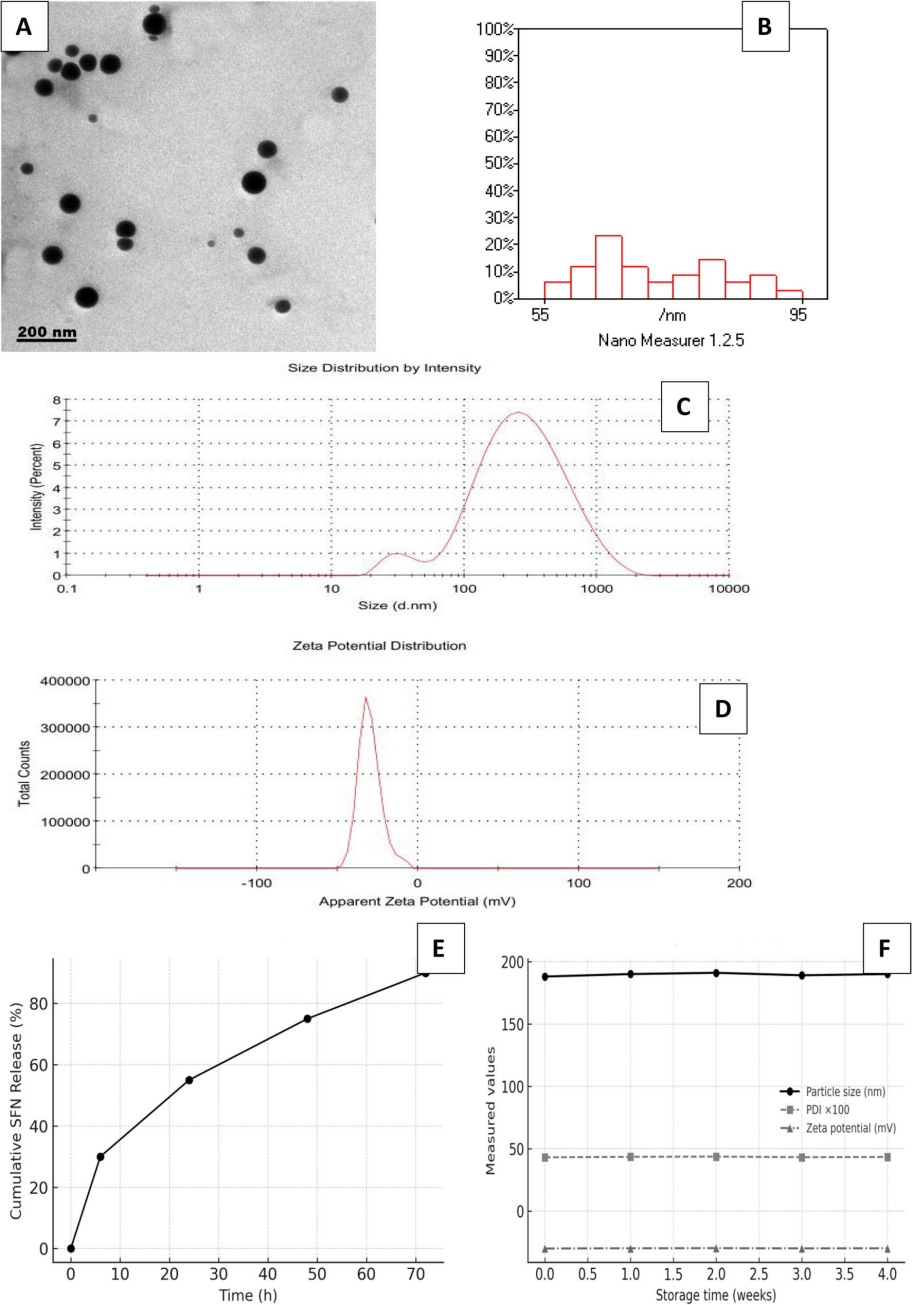
Characterization of SFN-loaded nanoliposomes: (**A**) TEM image showing nearly spherical particles with uniform morphology; (**B**) particle size distribution histogram indicating; (**C**) zeta size distribution by intenstiy; and (**D**) zeta potential distribution. In vitro cumulative release profile of SFN-NLPs (**E**).Storage stability of SFN-NLPs at 4 °C (**F**)

As visualized by transmission electron microscopy (TEM), SFN-NLPs displayed a spherical shape with consistent morphology (Fig. 2A), having diameters of approximately 55–95 nm (Fig. 2B). In contrast, DLS analysis yielded a hydrodynamic diameter of 188 nm, consistent with the larger size typically observed due to solvation effects. The polydispersity index (PDI) was calculated as 0.431, reflecting a moderately heterogeneous particle population (Fig. 2C). A zeta potential of − 30 mV further validated the nanoparticles’ stability, suggesting their suitability for biological applications (Fig. 2D). The hydrodynamic diameter measured by DLS (188 nm) is larger than the size observed by TEM (55–95 nm). This disparity is due to the hydration shell surrounding the nanoliposomes in aqueous solution, as well as the possible aggregation of vesicles in solution, which affects DLS measurements. In contrast, TEM provides the dry-state particle size, excluding the hydration layer, resulting in smaller apparent sizes. Additionally, the relatively high PDI (0.431) indicates a heterogeneous size distribution, further contributing to the observed differences. The SFN-NLPs achieved a loading content of 9.32% with an encapsulation efficiency of 79.41%, demonstrating successful drug incorporation. Along with their favorable physicochemical features, the formulation parameters indicate good stability and support its suitability for enhancing SFN delivery and bioavailability. Furthermore, the in vitro release profile of SFN-NLPs exhibited a biphasic pattern characterized by an initial burst release of approximately 25–30% within the first 6 h, followed by a sustained and gradual release up to 72 h, indicating prolonged drug retention within the lipid bilayer (Fig. 2E). The storage stability assessment revealed negligible changes in particle size, PDI, and zeta potential values over four weeks at 4 °C, with minimal drug leakage (< 5%), confirming the physicochemical stability of the nanoliposomes under refrigerated conditions (Fig. 2F).

### Changes in cell volume, viability, body weight and the mean survival time

**Fig. 3:**
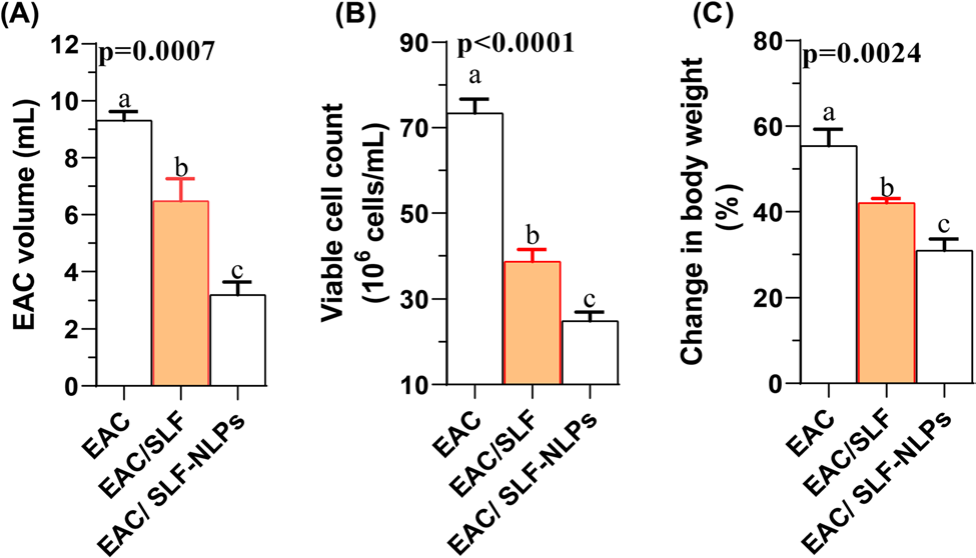
Changes in cell volume (A), viability (B), and body weight (C) in Ehrlich Ascites Carcinoma (EAC)-bearing mice treated with SFN-loaded nanoliposomes or free SFN. EAC: Inoculated with Ehrlich Ascites Carcinoma (EAC) cells (0.2 mL); EAC/SFN: SFN (50 mg/kg body weight) + EAC cells (0.2 mL); EAC/SFN-NLPs: SFN-loaded nanoliposomes (50 mg/kg body weight) + EAC cells (0.2 mL). Data are expressed as mean ± SE. Values in the same row with different superscript letters (**a**, **b**, **c**) differ significantly (*p* < 0.05)

**Fig. 4:**
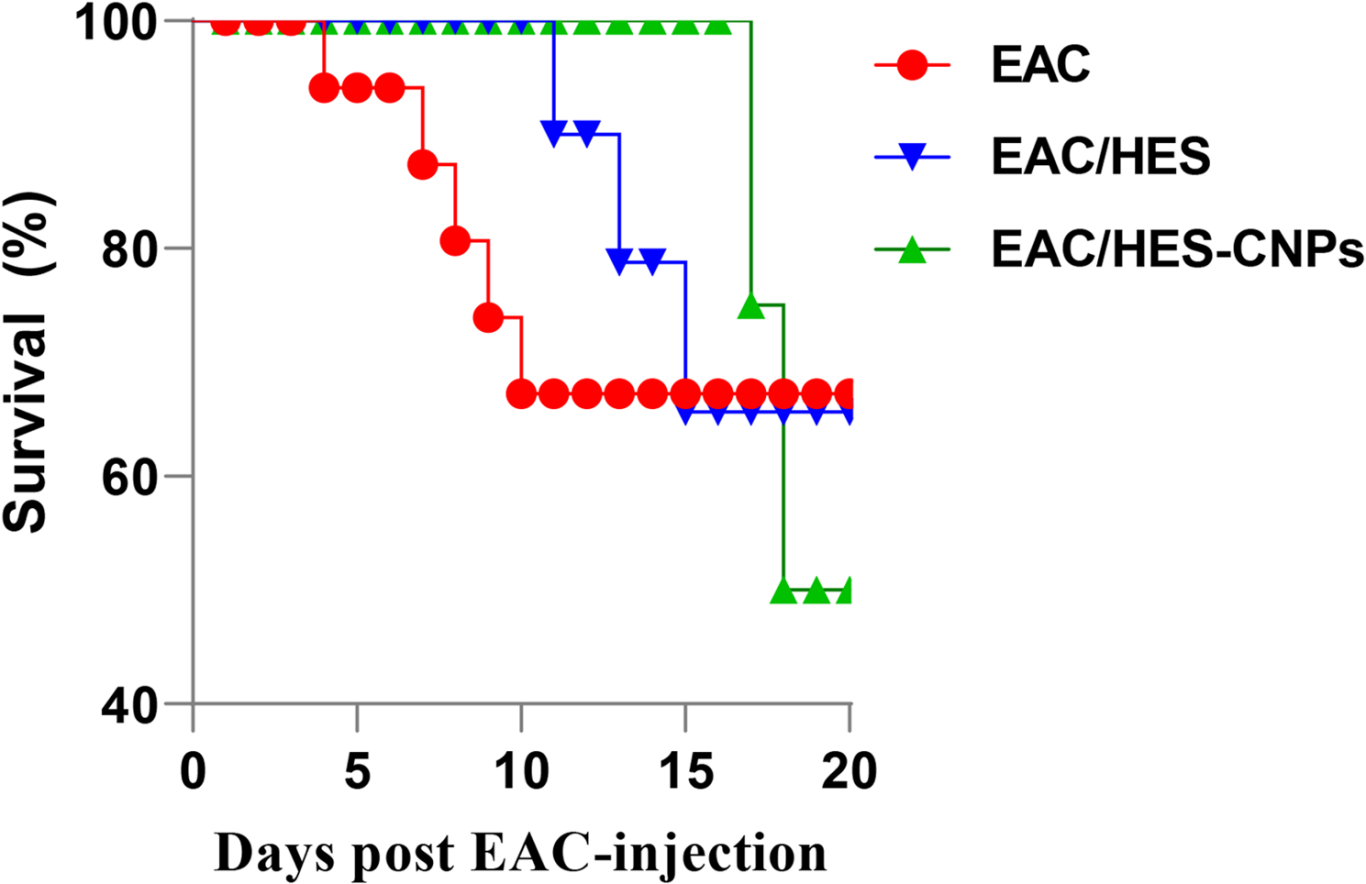
Kaplan–Meier survival curves showing the improved survival of EAC-bearing mice following SFN and SFN-NLPs treatment

Figure 3 illustrates that both crude SFN or SFN-NLPs treatments produced a significant reduction in ascitic fluid accumulation and viable cell numbers in EAC-bearing mice, with the most pronounced effect achieved by SFN-NLPs (*p* < 0.05). Untreated EAC group displayed the greatest body weight gain, whereas administration of SFN-NLPs effectively minimized weight increase, producing a more substantial effect than crude SFN (*p* < 0.05). Survival analysis revealed that cotreatment with crude SFN or SFN-NLPs significantly prolonged survival compared with untreated EAC-bearing mice. The SFN-NLPs group showed the most pronounced improvement, reducing the hazard of death by approximately 32% relative to controls (HR = 0.68, *p* = 0.09). Untreated mice survived an average of 7 days, whereas treatment with SFN extended survival to 13 days, and SFN-NLPs further prolonged it to 17.5 days. These findings highlight the enhanced therapeutic efficacy of the nanoliposomal formulation over the free compound (Fig. 4; Table 4).

**Table 4:** Survival analysis of EAC-bearing mice treated with SFN and SFN-NLPs using Kaplan–Meier and log-rank (Mantel–Cox) tests

Groups	Median (days)	95% CI	Events (*n*)	Log-rank vs. EAC (*p*-value)	Hazard ratio (vs. EAC)	95% CI (HR)
EAC	7	---	5	---	1	---
EAC/SFN	13	(11–15)	3	0.45	0.89	0.45–1.75
EAC/SFN-NLPs	17.5	(17–18)	2	0.09	0.68	0.33–1.38

*EAC *Inoculated with Ehrlich Ascites Carcinoma (EAC) cells (0.2 mL), *EAC/SFN *SFN (50 mg/kg body weight) + EAC cells (0.2 mL), *EAC/SFN-NLPs *SFN-loaded nanoliposomes (50 mg/kg body weight) + EAC cells (0.2 mL), *HR *hazard ratio, *CI* confidence interval

### Liver function and serum protein profile

Table 5 summarizes the alterations in serum biochemical parameters related to liver function following treatment with either crude SFN or SFN-NLPs in EAC bearing mice. In this study, the levels of total protein, albumin and globulin were significantly decreased in EAC-bearing mice compared with the control group (*p* < 0.05). The oral administration of SFN-NLPs significantly restored these levels (*p* < 0.05), bringing them close to those of the normal control group (*p* > 0.05). Regarding liver enzymes, the activities of LDH, ALP, ALT, and AST were significantly elevated following EAC injection. Administration of either form of SFN significantly reduced these enzyme activities, with the SFN-NLPs group showing no significant differences compared with the negative control group (*p* > 0.05).

**Table 5:** Effects of SFN-loaded nanoliposomes and free SFN on liver functions in Ehrlich Ascites carcinoma-bearing mice

Parameters	Control	SFN	SFN-NLPs	EAC	EAC/SFN	EAC/SFN-NLPs	*p*-value
TP (g/dL)	6.62 ± 0.41^a^	6.69 ± 0.44^a^	6.71 ± 0.39^a^	3.74 ± 0.08^b^	3.41 ± 0.21^b^	5.79 ± 0.35^a^	0.0001
Alb (g/dL)	3.65 ± 0.20^a^	3.74 ± 0.18^a^	3.77 ± 0.13^a^	1.84 ± 0.07^b^	2.01 ± 0.25^b^	3.19 ± 0.27^a^	0.0005
Glo (g/dL)	2.97 ± 0.21^a^	2.95 ± 0.26^a^	2.94 ± 0.26^a^	1.90 ± 0.01^b^	1.40 ± 0.04^b^	2.60 ± 0.08^a^	0.0001
LDH (U/L)	336.27 ± 3.24^c^	335.26 ± 2.83^c^	334.84 ± 3.11^c^	484.61 ± 5.41^a^	378.16 ± 5.45^b^	344.57 ± 3.79^c^	< 0.0001
ALP (U/L)	53.56 ± 4.58^c^	52.16 ± 5.07^c^	51.74 ± 4.70^c^	121.23 ± 6.32^a^	94.69 ± 5.11^b^	65.58 ± 5.93^c^	< 0.0001
ALT (U/L)	41.62 ± 3.93^c^	40.39 ± 2.81^c^	39.85 ± 3.39^c^	93.62 ± 5.81^a^	73.61 ± 4.06^b^	48.16 ± 4.33^c^	< 0.0001
AST (U/L)	59.20 ± 6.33^cd^	53.74 ± 2.29^d^	52.09 ± 3.04^d^	131.36 ± 7.41^a^	101.25 ± 5.11^b^	73.47 ± 7.13^c^	0.0002

*TP* Total protein, *Alb *Albumin, *Glo *Globulin, *LDH *Lactate dehydrogenase, *ALP *Alkalinephosphatase, *ALT *Alanine transaminase, *AST *Aspartate transaminase, *SFN *sulforaphane (50 mg/kgbody weight), *SFN-NLPs *sulforaphane-loaded nanoliposomes (50 mg/kg body weight), *EAC*Inoculated with Ehrlich Ascites Carcinoma (EAC) cells (0.2 mL), *EAC/SFN *SFN (50 mg/kg bodyweight) + EAC cells (0.2 mL), *EAC/ SFN-NLPs *SFN-loaded nanoliposomes (50 mg/kg body weight) +EAC cells (0.2 mL)

Data are expressed as mean ± SE. Values in the same row with differentsuperscript letters (a, b, c, d) differ significantly (*p* < 0.05)

### Tumor-associated biomarkers

Table 6 presents the changes in tumor-associated biomarkers. A significant increase was observed in all measured biomarkers, including AFP, CEA, CA19-9, CA-125, and CA15-3, following the injection of EAC cells (*p* < 0.05). Treatment with either SFN-NLPs or free SFN significantly reduced these elevated levels (*p* < 0.05). Notably, the levels of AFP, CEA, CA-125, and CA15-3 were significantly lower in the EAC/SFN-NLPs group compared with the EAC/SFN group, while no significant difference was observed between the two treatment forms for CA19-9 (*p* > 0.05). Furthermore, no significant differences were detected between the negative control group and the EAC/SFN-NLPs group for AFP and CEA biomarkers (*p* > 0.05).

**Table 6:** Effects of SFN-loaded nanoliposomes and free SFN on tumor-associated biomarkers in Ehrlich ascites carcinoma-bearing mice

Parameters	Control	SFN	SFN-NLPs	EAC	EAC/SFN	EAC/SFN-NLPs	*p*-value
AFP (ng/mL)	3.51 ± 0.26^c^	3.53 ± 0.22^c^	3.48 ± 0.19^c^	33.19 ± 4.93^a^	18.62 ± 2.28^b^	9.37 ± 1.11^c^	< 0.0001
CEA (ng/mL)	2.12 ± 0.19^c^	1.98 ± 0.18^c^	1.87 ± 0.21^c^	13.26 ± 2.12^a^	8.16 ± 1.39^b^	4.28 ± 0.83^c^	< 0.0001
CA19-9 (U/mL)	12.32 ± 1.47^c^	11.21 ± 1.66^c^	12.09 ± 2.45^c^	49.32 ± 5.74^a^	32.74 ± 3.96^b^	23.96 ± 2.37^b^	0.0001
CA-125 (U/mL)	7.41 ± 0.79^d^	6.29 ± 0.67^d^	6.37 ± 0.37^d^	53.91 ± 5.12^a^	41.12 ± 4.37^b^	18.66 ± 3.02^c^	< 0.0001
CA5-3 (U/mL)	10.12 ± 1.17^d^	9.65 ± 2.12^d^	9.55 ± 1.45^d^	58.61 ± 4.37^a^	38.21 ± 2.48^b^	25.36 ± 3.94^c^	0.0006

*AFP* alpha-fetoprotein, *CEA *carcinoembryonic antigen, *CA19-9 *cancer antigen 19-9, *CA-125*cancer antigen 125, *CA15-3* cancer antigen 15-3, *SFN *sulforaphane (50 mg/kg body weight), *SFNNLPs*sulforaphane-loaded nanoliposomes (50 mg/kg body weight), *EAC *Inoculated with EhrlichAscites Carcinoma (EAC) cells (0.2 mL), *EAC/SFN *SFN (50 mg/kg body weight) + EAC cells (0.2mL), *EAC/ SFN-NLPs *SFN-loaded nanoliposomes (50 mg/kg body weight) + EAC cells (0.2 mL)

Data are expressed as mean ± SE. Values in the same row with different superscript letters (a, b, c, d) differ significantly (*p* < 0.05)

### Expression profiles of inflammation related genes

**Fig. 5:**
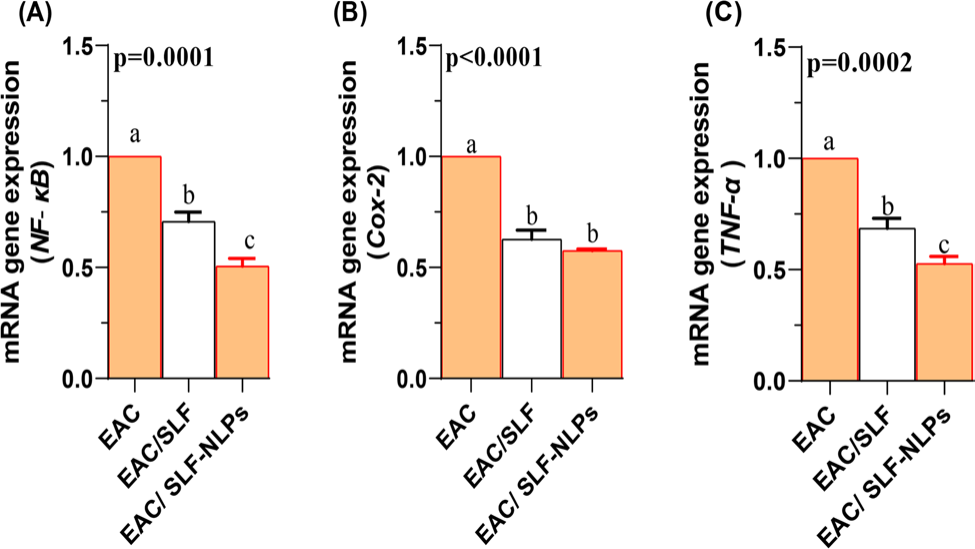
Expression of inflammation-related genes in Ehrlich ascites carcinoma (EAC)-bearing mice treated with sulforaphane-loaded nanoliposomes (SFN-NLPs) or free sulforaphane (SFN). Panels show: (A) NF-κB (nuclear factor kappa B), (B) Cox-2 (cyclooxygenase-2), and (C) TNF-α (tumor necrosis factor-alpha). Experimental groups: EAC, mice inoculated with EAC cells (0.2 mL); EAC/SFN, mice treated with free SFN (50 mg/kg body weight) plus EAC cells (0.2 mL); EAC/SFN-NLPs, mice treated with SFN-loaded nanoliposomes (50 mg/kg body weight) plus EAC cells (0.2 mL). Data are expressed as mean ± SE. Values in the same row with different superscript letters (a, b, c) differ significantly (p < 0.05)

Regarding the expression of inflammation-related genes, the transcription of NF-κB (Fig. 5A) was significantly alleviated following coadministration of either SFN-NLPs or free SFN, with NF-κB expression being significantly lower in the EAC/SFN-NLPs treated group compared with the EAC/SFN treated group (*p* < 0.05). Similarly, the expression of COX-2 (Fig. 5B) and TNF-α (Fig. 5C) were significantly elevated after EAC injection and significantly reduced in both treatment groups (*p* < 0.05), with no significant difference observed between EAC/SFN and EAC/SFN-NLPs treated groups for COX-2 expression (*p* > 0.05).

### Expression profiles of TP53, Bax, and Bcl2 genes and DNA fragmentation

**Fig. 6:**
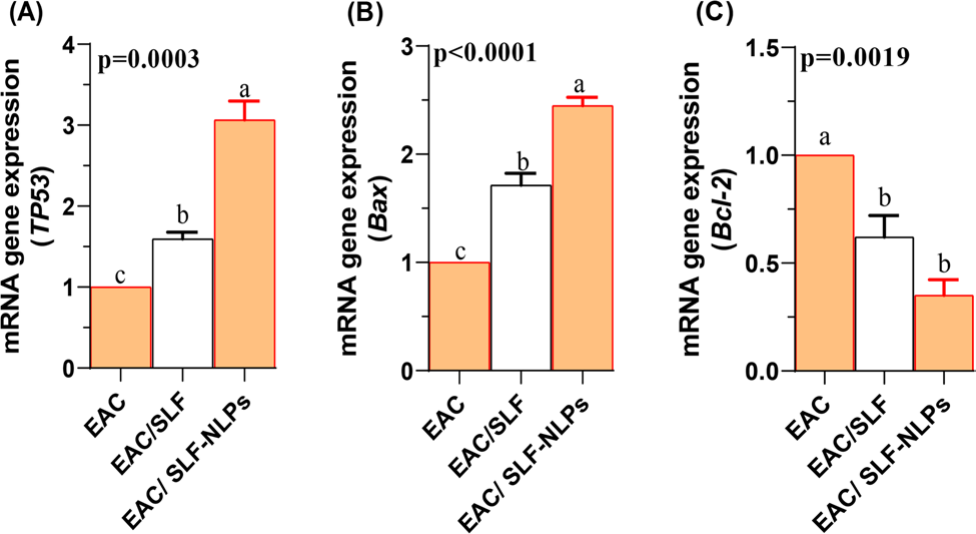
Expression profiles of apoptosis-related genes in Ehrlich ascites carcinoma (EAC)-bearing mice treated with sulforaphane (SFN) or SFN-loaded nanoliposomes (SFN-NLPs). Panels show: (A) TP53 (tumor protein 53), (B) Bax (Bcl-2-associated X protein), and (C) Bcl-2 (B-cell lymphoma 2). Experimental groups: EAC, mice inoculated with EAC cells (0.2 mL); EAC/SFN, mice treated with free SFN (50 mg/kg body weight) plus EAC cells (0.2 mL); EAC/SFN-NLPs, mice treated with SFN-loaded nanoliposomes (50 mg/kg body weight) plus EAC cells (0.2 mL). Data are expressed as mean ± SE. Values in the same row with different superscript letters (a, b, c) differ significantly (p < 0.05)

**Fig. 7:**
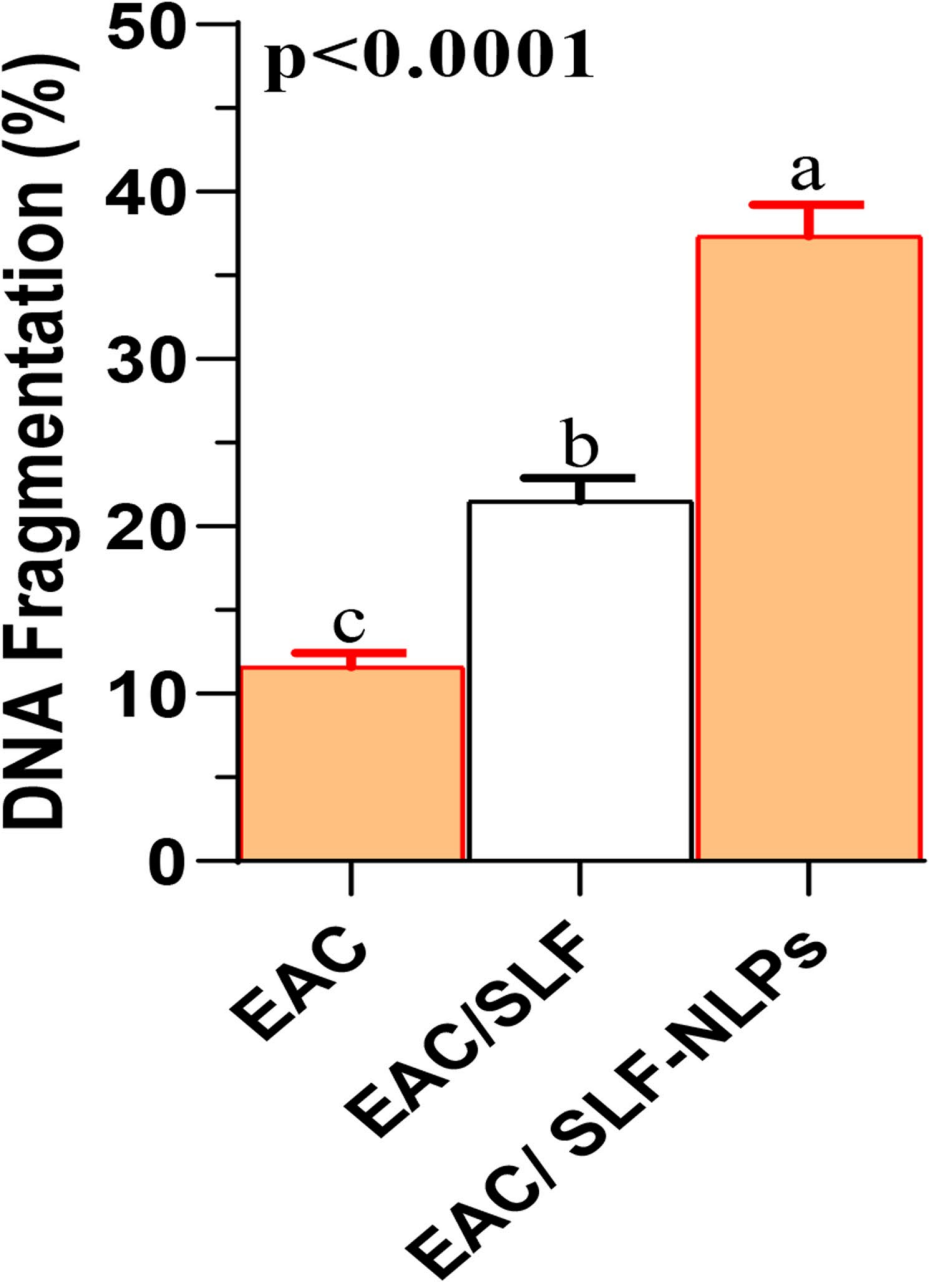
DNA fragmentation (%) in ehrlich ascites carcinoma (EAC)-bearing mice treated with SFN-loaded nanoliposomes or free sulforaphane. SFN: sulforaphane (50 mg/kg body weight); SFN-NLPs: sulforaphane-loaded nanoliposomes (50 mg/kg body weight); EAC: Inoculated with Ehrlich Ascites Carcinoma (EAC) cells (0.2 mL); EAC/SFN: SFN (50 mg/kg body weight) + EAC cells (0.2 mL); EAC/SFN-NLPs: SFN-loaded nanoliposomes (50 mg/kg body weight) + EAC cells (0.2 mL). Data are expressed as mean ± SE. Values in the same row with different superscript letters (**a**, **b**, **c**) differ significantly (*p* < 0.05)

In this study, co-treatment by either free SFN or SFN-NLPs significantly increased the expression of both TP53 (Fig. 6A) and Bax (Fig. 6B), with the highest expression levels detected in the EAC/SFN-NLPs-treated group (*p* < 0.05). Conversely, the expression of Bcl-2 (Fig. 6C) was significantly upregulated following EAC injection, whereas treatment with both SFN forms significantly downregulated its expression (*p* < 0.05). No significant difference was observed between the EAC/SFN and EAC/SFN-NLPs treated groups regarding Bcl-2 suppression (*p* > 0.05). With regard to DNA fragmentation, its percentage was significantly elevated in EAC-bearing mice but was markedly reduced following treatment. The reduction was more pronounced in the EAC/SFN-NLPs-treated group compared with the EAC/SFN-treated group (*p* < 0.05; Fig. 7).

### Redox status

**Fig. 8:**
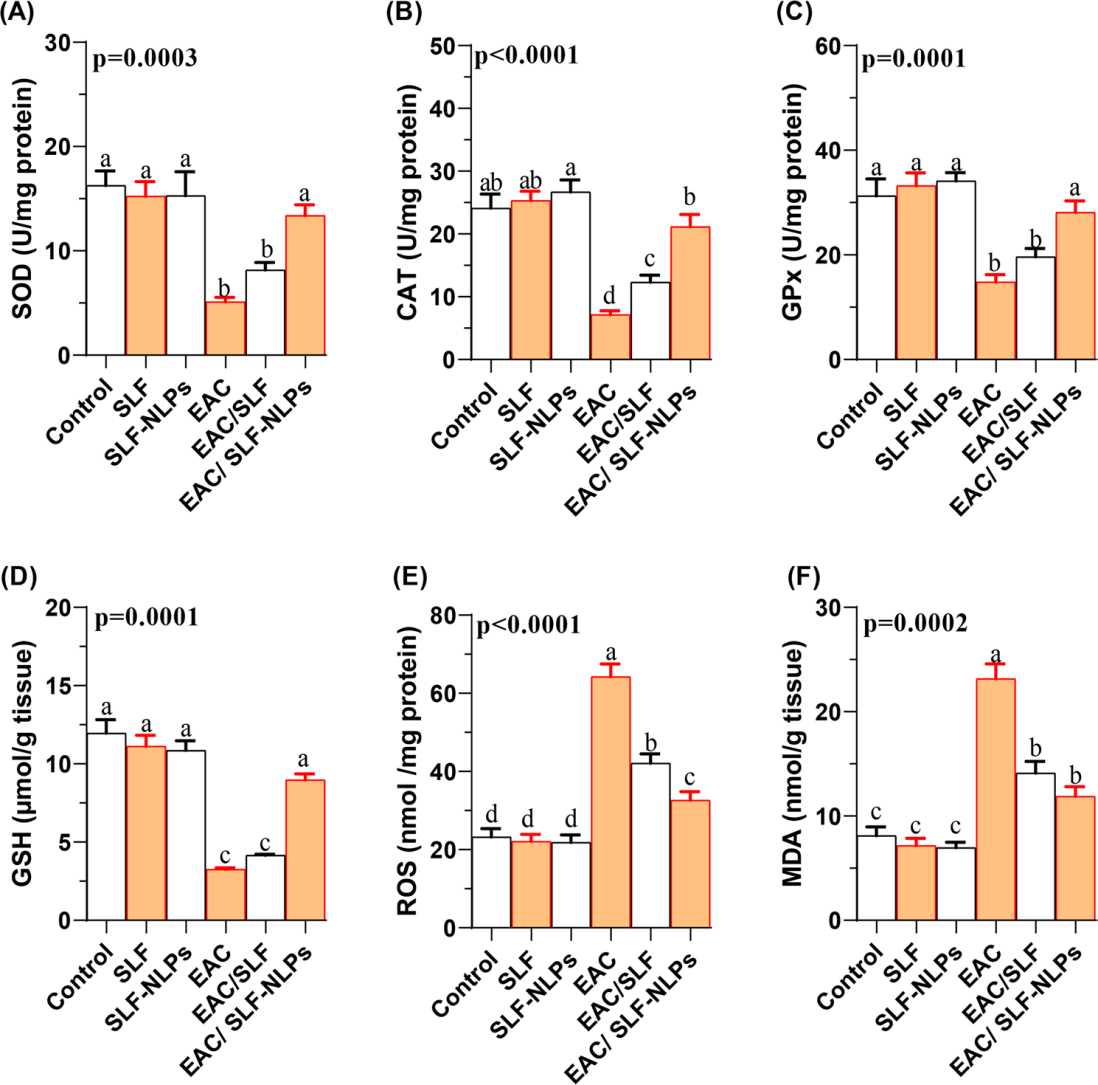
Hepatic antioxidant and oxidative stress markers in Ehrlich ascites carcinoma (EAC)-bearing mice treated with sulforaphane (SFN) or SFN-loaded nanoliposomes (SFN-NLPs). Panels show: (A) SOD (superoxide dismutase), (B) CAT (catalase), (C) GPx (glutathione peroxidase), (D) GSH (glutathione), (E) ROS (reactive oxygen species), and (F) MDA (malondialdehyde). Experimental groups: EAC, mice inoculated with EAC cells (0.2 mL); EAC/SFN, mice treated with free SFN (50 mg/kg body weight) plus EAC cells (0.2 mL); EAC/SFN-NLPs, mice treated with SFN-loaded nanoliposomes (50 mg/kg body weight) plus EAC cells (0.2 mL). Data are expressed as mean ± SE. Values in the same row with different superscript letters (a, b, c) differ significantly (p < 0.05)

With respect to hepatic redox status, the present results demonstrated a significant reduction in the activities of major antioxidant enzymes, including SOD (Fig. 8A), CAT (Fig. 8B), and GPx (Fig. 8C), in the liver tissues of EAC-bearing mice (*p* < 0.05). Co-treatment with either free SFN or SFN-NLPs significantly restored these enzyme activities (*p* < 0.05), with no significant differences observed between the negative control group and the EAC/SFN-NLPs-treated group (*p* > 0.05). Similarly, GSH levels were markedly reduced following EAC injection but significantly increased in the EAC/SFN-NLPs group (*p* < 0.05). In contrast, no significant differences were found between the EAC and EAC/SFN groups (*p* > 0.05; Fig. 8D). Regarding oxidative stress biomarkers, reactive oxygen species (ROS; Fig. 8E) were significantly elevated in EAC-bearing mice. Treatment with both SFN and SFN-NLPs significantly reduced ROS levels, with the reduction being more pronounced in the SFN-NLPs group compared to the SFN group (*p* < 0.05). Additionally, malondialdehyde (MDA; Fig. 8F), a marker of lipid peroxidation, was significantly increased in the EAC group but significantly decreased following the treatment by either SFN or SFN-NLPs, with no significant difference detected between EAC/SFN and EAC/SFN-NLPs treated groups (*p* > 0.05).

### Systemic inflammation

**Fig. 9:**
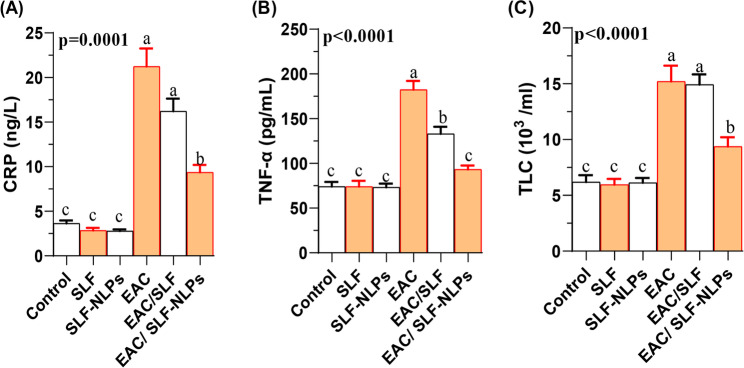
Systemic inflammation in Ehrlich ascites carcinoma (EAC)-bearing mice treated with sulforaphane (SFN) or SFN-loaded nanoliposomes (SFN-NLPs). Panels show: (A) CRP (C-reactive protein), (B) TNF-α (tumor necrosis factor-alpha), and (C) TLC (total leukocyte count). Experimental groups: EAC, mice inoculated with EAC cells (0.2 mL); EAC/SFN, mice treated with free SFN (50 mg/kg body weight) plus EAC cells (0.2 mL); EAC/SFN-NLPs, mice treated with SFN-loaded nanoliposomes (50 mg/kg body weight) plus EAC cells (0.2 mL). Data are expressed as mean ± SE. Values in the same row with different superscript letters (a, b, c) differ significantly (p < 0.05)

In this study, the concentrations of CRP (Fig. 9A), TNF-α (Fig. 9B), and TLC (Fig. 9C) were significantly elevated in EAC-bearing mice compared with the control s (*p* < 0.05). Treatment with either free SFN or SFN-NLPs significantly reduced these elevated levels, with the most pronounced reduction detected in the EAC/SFN-NLPs-treated mice. No significant difference was observed between the EAC group and the EAC/SFN treated group with respect to CRP concentration. Regarding TLC, it was significantly increased in EAC-bearing mice but markedly decreased following SFN-NLPs administration (*p* < 0.05).

### Flowcytometry studies

**Fig. 10:**
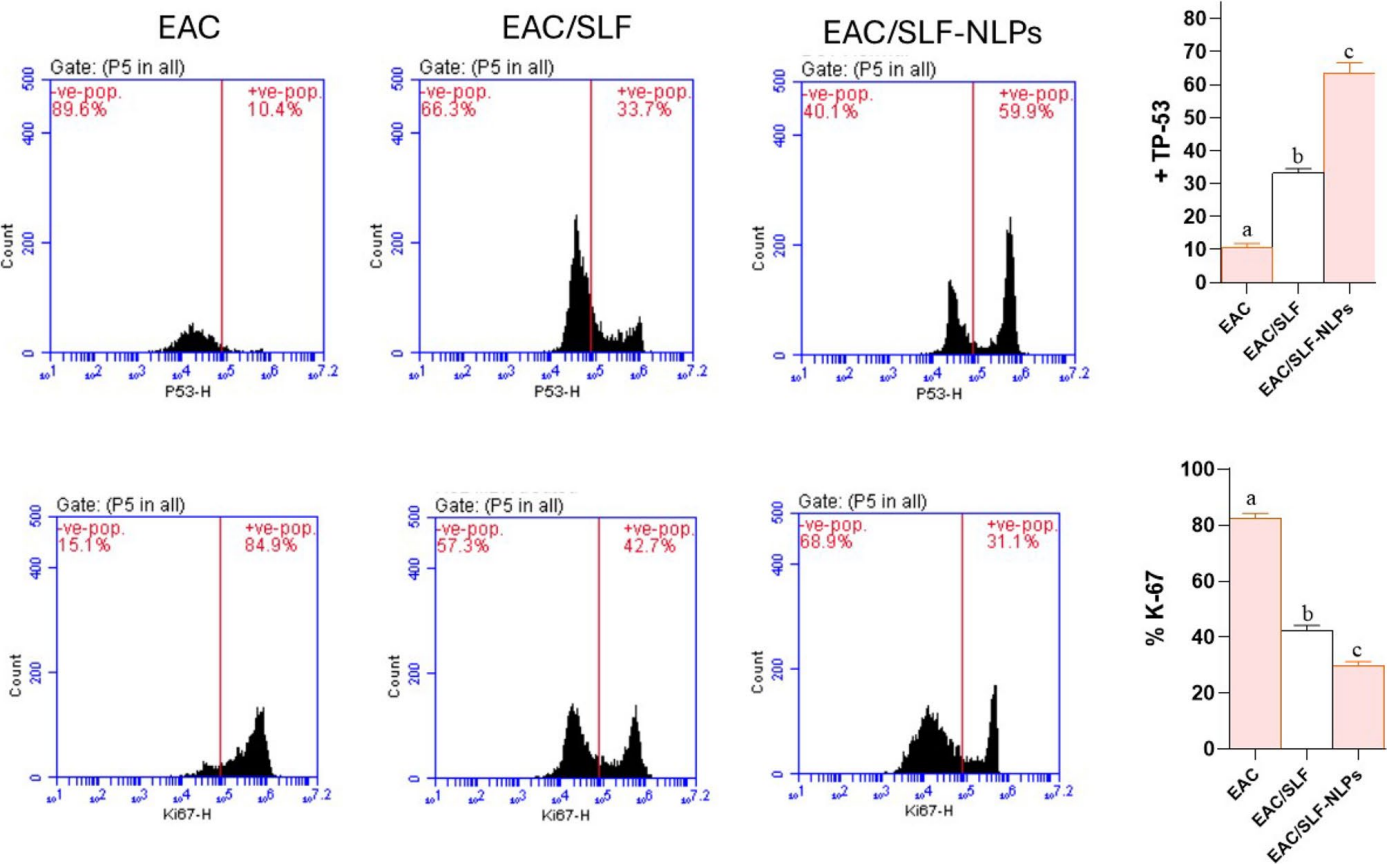
Flow cytometry analysis showing the expression levels of TP-53 (top row), Ki-67 (bottom row), and respective quantification graphs (right panels). The histograms depict the distribution of positive and negative populations for each marker across different experimental conditions: EAC, EAC/SFN, and EAC/SFN-NLPs. The bar graphs illustrate the percentage of TP-53 positivity (top right) and Ki67 positivity (bottom right) in each condition, with significant differences (*p*-value < 0.05). Data represents the mean ± SD of three independent experiments

The flow cytometry analysis indicated significant differences in the expression profiles of p53 and Ki-67 across the experimental groups. The EAC group exhibited a low proportion of p53-positive cells (10.5 ± 2.8%) and high Ki-67-positive cells (82.4 ± 4.3%), indicating low activity of the tumor suppressor and accelerated cell division. In contrast, the EAC/SFN and EAC/SFN-NLPs groups demonstrated a notable increase in p53-positive cells (33.2 ± 2.9% and 63.4 ± 7.1%, respectively) and marked decline in Ki-67 positivity (42.2 ± 3.5% and 29.6 ± 3.6%, respectively). Quantitative analysis supported these findings, demonstrating a significant increase in P-53 and notable decrease in Ki-67 expression in the EAC/NLPs group compared to the EAC and EAC/SFN groups (*p* < 0.05) (Fig. 10).

### Histopathological results

**Fig. 11:**
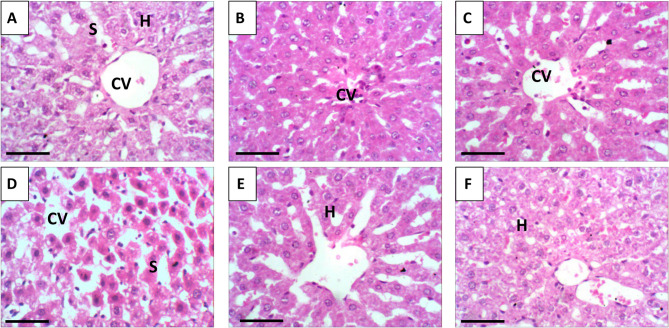
Representative photomicrographs of liver sections from the control and different experimental groups. **A** control group; (**B**) SFN: sulforaphane (50 mg/kg body weight); (**C**) SFN-NLPs: sulforaphane-loaded nanoliposomes (50 mg/kg body weight); (**D**) EAC: Inoculated with Ehrlich Ascites Carcinoma (EAC) cells (0.2 mL); (**E**) EAC/SFN: sulforaphane (50 mg/kg body weight) + EAC cells (0.2 mL); (**F**) EAC/SFN-NLPs: sulforaphane-loaded nanoliposomes (50 mg/kg body weight) + EAC cells (0.2 mL). (CV) center vein, (S)sinusoids, (H)hepatocytes. All image at = 400x. scale bar 50 μm

**Fig. 12:**
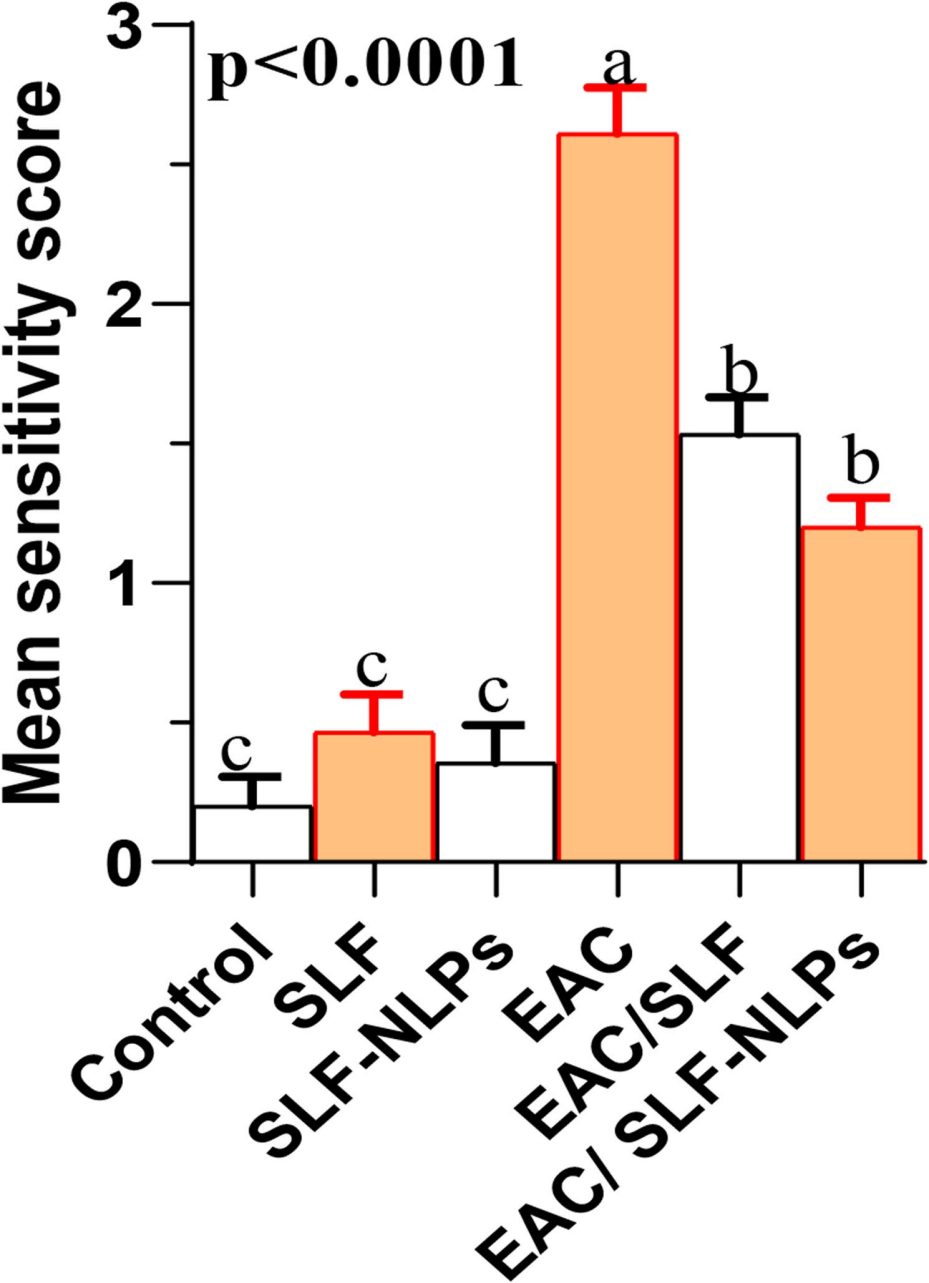
Comparison of severity scores of hepatic tissue injury in control and treated groups. SFN: sulforaphane (50 mg/kg body weight); SFN-NLPs: sulforaphane-loaded nanoliposomes (50 mg/kg body weight); EAC: Inoculated with Ehrlich Ascites Carcinoma (EAC) cells (0.2 mL); EAC/SFN: SFN (50 mg/kg body weight) + EAC cells (0.2 mL); EAC/SFN-NLPs: SFN-loaded nanoliposomes (50 mg/kg body weight) + EAC cells (0.2 mL). Data are expressed as mean ± SE. Values in the same row with different superscript letters (**a**, **b**, **c**) differ significantly (*p* < 0.05)

Histopathological examination of liver tissue (Fig. 11A–C) confirmed that treatment with either free SFN or SFN-NLPs maintained normal hepatic morphology closely resembling the normal histoarchitecture observed in control animals, indicating their safety and absence of hepatotoxic effects. The examined liver sections maintained normal hepatic architecture, showing intact central regions, organized lobules, and hepatocytes characterized by central nuclei, and polygonal/hexagonal morphology. Striking pathological changes were observed in the livers of EAC bearing mice, manifesting as dilated sinusoids, nuclear necrosis, and pronounced vacuolar degeneration of hepatocytes (Fig. 11D). Remarkably, treatment with SFN or SFN-NLPs in EAC-bearing mice led to notable restoration of liver histology, as evidenced by hepatic cords aligned around central veins and re-established lobular arrangement (Fig. 11E–F). The EAC-treated mice exhibited a marked increase in the histopathological severity score compared to the control, SFN-, or SFN-NLPs-administered groups. In contrast, co-administration of SFN or SFN-NLPs with EAC significantly attenuated the EAC-induced histopathological damage (Fig. 12).

### Ultra structural changes

**Fig. 13:**
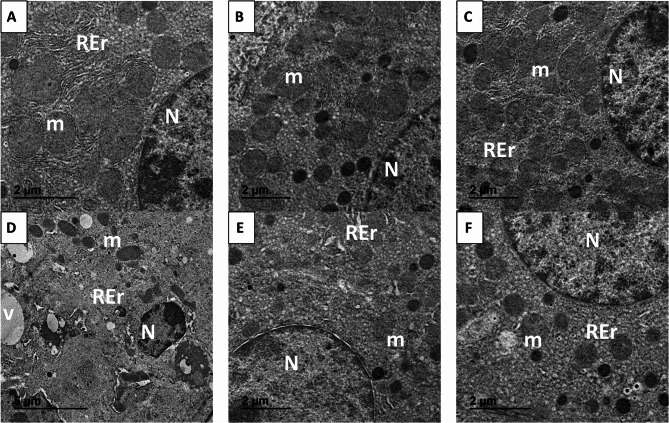
Representative transmission electron micrographs of liver tissues from the control and different experimental groups. **A** control group; **B**) SFN: sulforaphane (50 mg/kg body weight); **C**) SFN-NLPs: sulforaphane-loaded nanoliposomes (50 mg/kg body weight); **D**) EAC: Inoculated with Ehrlich Ascites Carcinoma (EAC) cells (0.2 mL); **E**) EAC/SFN: sulforaphane (50 mg/kg body weight) + EAC cells (0.2 mL); **F**) EAC/SFN-NLPs: sulforaphane-loaded nanoliposomes (50 mg/kg body weight) + EAC cells (0.2 mL). (N) nucluse, (m)mitochondria, (REr) rough endoplasmic reticulum, (V) cytoplasm vacuolated

Transmission electron micrographs (Fig. 13A–C) indicated that hepatocytes from the negative control and both treatment groups (SFN and SFN-NLPs) retained normal architecture, with preserved nuclear shape and nucleoli, structurally sound mitochondria, and well-organized rough endoplasmic reticulum, confirming the absence of cytotoxic effects and supporting the biocompatibility and safety of both SFN and SFN-NLPs. Hepatocytes from EAC-treated mice showed necrotic features, such as cytoplasmic degeneration, condensed chromatin, vacuolation, expanded endoplasmic reticulum, disrupted mitochondria with fragmented cristae, and distorted nuclei (Fig. 13D). The hepatic ultrastructure of mice treated with EAC plus SFN (Fig. 13E) or EAC plus SFN-NLPs (Fig. 13F) exhibited restored ultrastructural features, including slight vacuolation, well-preserved organelles, and minimal mitochondrial degeneration, demonstrating notable structural enhancement.

### Multivariable analysis

**Fig. 14:**
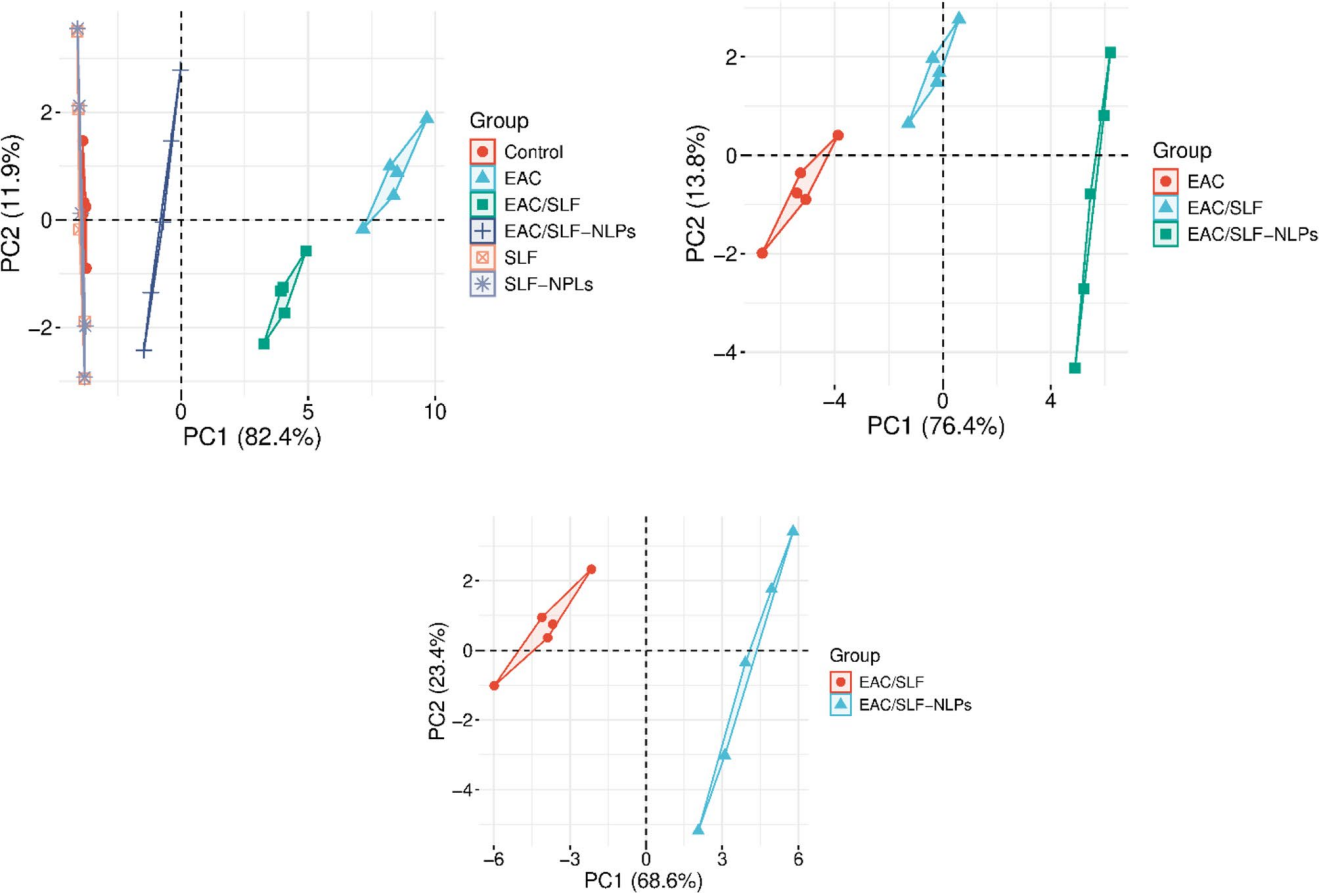
Principal component analysis (PCA) score plots illustrating the metabolic or molecular profile differentiation among experimental groups. Each plot displays the distribution of experimental groups [control group; SFN: sulforaphane (50 mg/kg body weight); SFN-NLPs: sulforaphane-loaded nanoliposomes (50 mg/kg body weight); EAC: Inoculated with Ehrlich Ascites Carcinoma (EAC) cells (0.2 mL); EAC/SFN: sulforaphane (50 mg/kg body weight) + EAC cells (0.2 mL); EAC/SFN-NLPs: sulforaphane-loaded nanoliposomes (50 mg/kg body weight) + EAC cells (0.2 mL)] across the first two principal components. The PCA demonstrates clear separation between the control and EAC groups, Notably, the EAC/SFN-NLPs group clusters closer to the control, suggesting a more effective restoration of metabolic homeostasis compared to free SFN, emphasizing the therapeutic potential of nanoliposome encapsulation for improved bioactivity

PCA indicates that the experimental groups are clustered according to the principal component. The initial PCA plot demonstrates a clear separation between the control and EAC groups along PC1. The EAC group exhibits a more dispersed distribution, suggesting notable metabolic or molecular abnormalities resulting from EAC inoculation. The EAC/SFN and EAC/SFN-NLPs groups exhibit an intermediate position, indicating partial regulation of EAC-related changes, with EAC-NLPs approaching the control group. The second PCA figure, accounting for 81.8% of the variance, indicates that SFN and SFN-NLPs alter the metabolic profile relative to the EAC baseline. The EAC/SFN-NLPs group exhibits greater divergence from the EAC group compared to the EAC/SFN group, indicating that nanoliposome-encapsulated SFN may offer superior modulation. The third PCA plot indicates that the EAC/SFN-NLPs group clusters distinctly from the EAC group, implying a unique metabolic profile linked to nanoliposome administration. PCA data indicates that both forms of SFN mitigate EAC-induced alterations, with nanoliposomes demonstrating a greater restorative effect (Fig. 14).

## Discussion

The EAC mouse model is a valuable tool for investigating tumor–host metabolic interactions [35]. EAC is an undifferentiated, aggressively proliferating, and transplantable tumor that responds strongly to chemotherapeutic interventions, making it a common choice for preclinical anticancer research [36, 37]. Chemotherapy is a primary treatment modality for cancer; however, its side effects and restricted efficacy have prompted increased interest in plant-derived compounds as promising anticancer candidates [10, 37]. SFN, a bioactive compound found in vegetables, counteracts cancer by multiple mechanisms, including cell cycle arrest, carcinogen detoxification, and inhibition of proliferation. Additionally, SFN can synergistically enhance the effectiveness of conventional chemotherapy [38]. SFN’s therapeutic potential is hindered by its poor stability and brief half-life in clinical settings [24].

Molecular docking studies indicate that SFN may positively interact with key proteins involved in inflammatory and apoptotic pathways, such as NF-κB, COX-2, and Bax, thereby supporting its proposed bioactivity in these processes. The identified hydrophobic interactions and hydrogen bonds indicate that SFN can effectively bind to active regions, potentially leading to efficient modulation of protein function. It should be noted that molecular docking serves as mechanistic support only, providing insights into possible interactions rather than direct proof of biological efficacy, and the selected binding sites were chosen based on their known involvement in relevant biological pathways. The in-silico findings provide valuable insights into potential molecular interactions; however, they are limited by the inherent simplifications of docking simulations and do not account for dynamic biological contexts, protein conformational flexibility, or in vivo bioavailability [39]. To verify these anticipated interactions and their biological significance, functional assays were conducted in the study for experimental confirmation.

Encapsulating SFN in liposomes significantly improves its stability, facilitates sustained release, and boosts its anticancer, anti-inflammatory, and antioxidant properties [28]. With high drug-loading capacity and additional stabilization through lipid–protein interactions, liposomes represent versatile biocompatible nanocarriers for multiple biomedical applications [40]. In this study, SFN-NLPs were developed to assess their anticancer efficacy in EAC- bearing mice relative to free SFN, with a focus on reducing oxidative stress, suppressing inflammation in liver tissue, and triggering apoptosis in carcinoma cells. The present results revealed that SFN-NLPs possess greater anticancer efficacy against EAC than unencapsulated SFN. They markedly enhanced cytotoxicity while exerting strong antioxidant, pro-apoptotic, and anti-inflammatory properties [41–43]. The combined actions of SFN-NLPs resulted in substantial inhibition of tumor growth and extended survival in treated mice. By comparison, untreated EAC-bearing mice experienced high mortality and poor survival, which can be attributed to tumor-induced inflammation. Such inflammatory responses may increase capillary permeability and impair both lymphatic and vascular function, leading to the buildup of protein-rich peritoneal fluid [44]. Tumor markers, including AFP, CEA, CA19-9, CA-125, and CA15-3, are routinely used for the diagnosis and surveillance of cancer [45]. The present results revealed that mice harboring EAC tumors displayed significantly elevated levels of these markers, suggesting both tumor aggressiveness and a high metastatic nature. SFN-NLPs administration significantly lowered these markers relative to free SFN, possibly through mechanisms involving tumor growth inhibition, reduction of inflammation, apoptosis promotion, and enhancement of antioxidant defenses, while the nanoparticle delivery system provided better stability, controlled release, and therapeutic efficacy [28, 46].

A microenvironment characterized by chronic inflammation can accelerate cancer onset and progression [47]. Such inflammation promotes tumorigenesis through diverse mechanisms, including DNA damage, tissue remodeling, immune suppression, and enhanced cellular proliferation [48]. The anti-inflammatory activities of SFN are well supported by multiple studies [14, 49]. NF-κB activation is a common feature in different cancers, promoting the expression of genes involved in pro-inflammatory cytokine synthesis, apoptosis resistance, cell adhesion, and growth factor signaling [50]. Inflammation inhibition is recognized as the sixth mechanism through which SFN impedes tumor progression. Prior studies have indicated that SFN achieves this primarily by modulating NF-κB and its downstream target COX-2 to achieve anti-inflammatory effects [14]. In this study, SFN-NLPs administration significantly suppressed the expression of NF- κB, COX-2, and TNF-α in carcinoma cells. Furthermore, SFN can boost cell-mediated immune responses in tumor-bearing mice, contributing to tumor growth suppression [51]. Chronic inflammation enhances cancer cell survival and resistance to apoptosis [50], highlights that SFN’s antitumor activity involves not only anti-inflammatory effects but also the reactivation of apoptotic pathways, thereby improving its overall efficacy. Previous investigations of SFN and other phytochemicals from cabbage have largely targeted classical apoptotic mechanisms, demonstrating the activation of p21, p53, Bax, and caspase-3, along with the downregulation of Bcl-2 in a variety of cancer cell lines [20, 21, 41]. In this study, SFN-NLP administration further corroborated previous findings by significantly enhancing apoptosis, as evidenced by the downregulation of the anti-apoptotic gene Bcl-2 and the upregulation of pro-apoptotic genes (TP53 and Bax) in tumor tissues. Complementary evidence from DNA fragmentation confirmed the apoptotic activity in cancer cells. Increased Bax expression or decreased Bcl-2 levels promotes mitochondrial cytochrome c release, which in turn activates caspase-9 and then caspase-3. Caspase-3 activation causes ICAD cleavage, allowing CAD to degrade DNA and produce the typical DNA fragmentation seen in apoptotic cells [52]. Beltagy et al. [41] observed that SFN induces DNA fragmentation in a laddering pattern, which is characteristic of apoptosis rather than necrosis. Similarly, our study demonstrated that SFN-NLPs administration significantly increased DNA fragmentation in EAC-bearing mice. Collectively, these results highlight SFN’s dual function in modulating inflammation and promoting apoptosis, underscoring its potential as a cancer therapeutic.

The liver, being both the largest and a highly vital organ, regulates a multitude of biochemical and physiological pathways. Natural dietary compounds were shown to protect the liver and preserve its architectures under various physiological and pathological conditions via their antioxidant and anti-inflammatory effects [53, 54]. SFN supports liver health through multiple mechanisms, including the protection from oxidative stress and hepatic inflammation, induction of detoxifying and antioxidant enzymes, and chemopreventive as well as anticancer properties [55]. Excessive accumulation of ascitic fluid in EAC-bearing mice led to pathological alterations that significantly impaired hepatocyte activity and overall hepatic integrity. In this study, serum levels of AST, ALT, LDH, and ALP were significantly elevated in EAC-bearing mice, In line with prior reports [56]. The observed elevation is associated with increased ROS and oxidative stress, resulting in lipid peroxidation, impair Ca²⁺ transport and storage in mitochondria, endoplasmic reticulum, and the plasma membrane, raise plasma Ca²⁺ concentrations, and culminate in cell death, leading to the release of AST, ALT, LDH, and ALP into the circulation [57, 58]. SFN-NLPs administration provided significant hepatocyte protection, evidenced by reductions in AST, ALT, LDH, and ALP levels along with elevated total serum protein and its fractions compared to untreated EAC mice. Overall, these results support the hepatoprotective role of SFN [59]. The histopathological analysis aligned with the biochemical results, revealing pronounced hepatic degeneration in untreated mice. Conversely, SFN-NLPs administration preserved liver morphology and ultrastructure, including properly aligned hepatocytes and organized hepatic cords, reflecting the antioxidant-mediated protective effect on hepatocyte function. Oxidative stress induced by EAC was evident in the liver tissues of affected mice, demonstrated by elevated MDA levels, a significant reduction in GSH content, and decreased activity of major antioxidant enzymes, including CAT, SOD, and GPx. The increase in MDA levels suggests enhanced lipid peroxidation, likely arising from ROS overproduction and accumulation of thiobarbituric acid-reactive substances, which collectively contribute to macromolecular damage within cells [16, 17, 60]. Elevated cholesterol levels in EAC-bearing mice have been correlated with increased lipid peroxidation [61]. Reduced erythrocyte GSH levels can impair the activity of antioxidant enzymes such as CAT, SOD, and GPx. This reduction in antioxidant defense may weaken the immune system, thereby increasing susceptibility to cancer [62]. Additionally, the loss of mitochondrial Mn-SOD within tumor cells may contribute to diminished SOD activity and further hepatic antioxidant depletion [63]. In this study, SFN-NLPs treatment significantly alleviated oxidative damage by elevating CAT, SOD, and GPx, activities, restoring GSH levels, and lowering MDA in hepatic tissue, showing greater efficacy than crude SFN. It was reported that SFN demonstrates antioxidant activity by reducing oxidative stress and protecting cells against cytotoxic injury. This activity primarily involves the activation of the transcription factor Nrf2, which governs the expression of cellular antioxidant enzymes and protective molecules [42].

Beyond alleviating hepatic oxidative damage, SFN-NLPs administration effectively modulate the systemic inflammation linked to EAC, underscoring their dual protective effects. EAC progression was associated with systemic inflammation, indicated by marked elevations in CRP, TNF-α, and leukocyte counts. CRP, a sensitive marker for both liver injury and systemic inflammation [64], is likely increased due to oxidative damage and amplified inflammatory signaling induced by EAC [65]. The observed leukocytosis may reflect responses to cellular and biochemical changes, tumor formation, or consequences of lipid peroxidation and lymphocyte depletion [66]. Oxidative stress-induced lipid peroxidation can enhance TNF-α levels by activating NF-κB, releasing MDA, and increasing pro-inflammatory cytokine production, which drives apoptosis, hepatocyte damage, and activation of hepatic stellate cells [67]. SFN-NLPs treatment markedly improved systemic inflammatory parameters, as evidenced by decreases in TNF-α, CRP, and leukocyte levels. These effects may result from SFN’s mitigation of EAC-induced oxidative stress, its tumor-regressive activity, and its inherent anti-inflammatory capabilities [14]. According to Treasure et al. [68], SFN mitigates inflammation through Nrf2 activation, which regulates immune mediators including MIF, inhibits NF-κB–driven cytokine production, boosts antioxidant defenses, and modulates inflammasome activity, ultimately curbing exaggerated immune responses and potentially reducing steroid requirements [69]. These observations suggest that SFN-NLPs administration strengthens antioxidant defenses in liver tissue while mitigating EAC-induced oxidative stress, supporting their role as a therapeutic approach for protecting against hepatic damage.

Recent studies indicate a significant association between the metabolic reprogramming of tumor microenvironment macrophages and oxidative stress and inflammation. Zhang et al. [70] demonstrated that the PKM2/HIF-1α axis regulates macrophage polarization and metabolic adaptability, influencing both pro- and anti-tumor immune responses. The findings indicate that SFN-NLPs may exert efficacy through the suppression of oxidative and inflammatory processes, thereby indirectly influencing macrophage activity and redox balance within the hepatic tumor microenvironment. The findings indicate that SFN-NLPs may enhance antioxidant defenses and address hepatic damage induced by EAC. These observations suggest that SFN-NLPs administration strengthens antioxidant defenses and mitigates inflammation in liver tissue while mitigating EAC-induced oxidative stress, supporting their role as a therapeutic approach for protecting against hepatic damage.

Notwithstanding these favorable outcomes, the study exhibited several limitations. The study was performed solely in a mouse model, which may not adequately represent the complexity of human cancers and their microenvironments, thereby restricting its therapeutic relevance. The study primarily examined molecular and biochemical parameters, neglecting long-term toxicity and off-target effects of nanoformulated SFN, which are essential for assessing safety and therapeutic viability. Moreover, only a single dose of SFN-NLPs was tested, without comparison to standard chemotherapeutic agents, which limits conclusions regarding relative efficacy. Additionally, the study did not include an empty nanoliposomes (Empty NLPs) control group, which prevents distinguishing the therapeutic effects of SFN from potential non-specific effects of the nanocarrier itself. Molecular docking indicated SFN interactions with target proteins; however, functional validation via in vivo or in vitro experiments is necessary to confirm these pathways. Finally, SFN-NLP bioavailability and systemic behavior are contingent upon pharmacokinetic factors such as absorption, distribution, metabolism, and excretion. Future studies examining the in vivo distribution and targeting mechanisms of SFN-NLPs would provide valuable insights into their therapeutic potential and organ-specific delivery. Future studies must address these limitations to comprehensively evaluate the anticancer therapeutic potential and safety of SFN-NLPs.

## Conclusion

Loading SFN onto liposomal nanoparticles enhanced its stability, increased its efficacy, and enabled sustained release, making it more effective than unprocessed SFN. Administration of SFN-NLPs at 50 mg/kg body weight significantly suppressed tumor growth by modulating inflammation and promoting apoptosis in tumor cells. Moreover, SFN-NLPs preserved liver integrity by reducing systemic inflammation and counteracting oxidative stress, as evidenced by decreased levels of lipid peroxides and reactive oxygen species, along with enhanced activities of key antioxidant enzymes. This treatment provided superior protection against both histopathological and ultra-structural liver damage, reflecting its antioxidant, anti-inflammatory, and anti-apoptotic properties. These effects were further supported by molecular docking analysis, which revealed a strong binding affinity of SFN to proteins involved in antioxidant defense, inflammation regulation, and apoptosis.

## Data Availability

The data supporting the findings of this study are available from the corresponding author upon reasonable request. No publicly archived datasets are applicable.
